# Gut Bacterial Community of the Xylophagous Cockroaches *Cryptocercus punctulatus* and *Parasphaeria boleiriana*

**DOI:** 10.1371/journal.pone.0152400

**Published:** 2016-04-07

**Authors:** Mercedes Berlanga, Carlos Llorens, Jaume Comas, Ricardo Guerrero

**Affiliations:** 1 Department of Microbiology and Parasitology, Faculty of Pharmacy, University of Barcelona, Barcelona, Spain; 2 Unity of Genomics. Scientific and Technological Centers, University of Barcelona (CCiTUB), Barcelona, Spain; 3 Biotechvana, Valencia, Spain; 4 Laboratory of Molecular Microbiology and Antimicrobials, Department of Pathology and Experimental Therapeutics, Faculty of Medicine, University of Barcelona-IDIBELL, Barcelona, Spain; 5 Barcelona Knowledge Hub, *Academia Europaea*, Barcelona, Spain; Free University of Bozen/Bolzano, ITALY

## Abstract

*Cryptocercus punctulatus* and *Parasphaeria boleiriana* are two distantly related xylophagous and subsocial cockroaches. *Cryptocercus* is related to termites. Xylophagous cockroaches and termites are excellent model organisms for studying the symbiotic relationship between the insect and their microbiota. In this study, high-throughput 454 pyrosequencing of 16S rRNA was used to investigate the diversity of metagenomic gut communities of *C*. *punctulatus* and *P*. *boleiriana*, and thereby to identify possible shifts in symbiont allegiances during cockroaches evolution. Our results revealed that the hindgut prokaryotic communities of both xylophagous cockroaches are dominated by members of four *Bacteria* phyla: *Bacteroidetes*, *Firmicutes*, *Proteobacteria*, and *Actinobacteria*. Other identified phyla were *Spirochaetes*, *Planctomycetes*, *candidatus* Saccharibacteria (formerly TM7), and *Acidobacteria*, each of which represented 1–2% of the total population detected. Community similarity based on phylogenetic relatedness by unweighted UniFrac analyses indicated that the composition of the bacterial community in the two species was significantly different (*P* < 0.05). Phylogenetic analysis based on the characterized clusters of *Bacteroidetes*, *Spirochaetes*, and *Deltaproteobacteria* showed that many OTUs present in both cockroach species clustered with sequences previously described in termites and other cockroaches, but not with those from other animals or environments. These results suggest that, during their evolution, those cockroaches conserved several bacterial communities from the microbiota of a common ancestor. The ecological stability of those microbial communities may imply the important functional role for the survival of the host of providing nutrients in appropriate quantities and balance.

## Introduction

Insects account for most of the richness of species of the animal clades on Earth. The associations between microorganisms and insects are widespread in nature [1]. For their insect hosts, bacteria can provide numerous benefits, such as specific nutritional complementation of a markedly imbalanced diet, protection from predators, parasites, and pathogens; and the promotion of mating and reproduction [2,3]. Termites (*Isoptera*), cockroaches, and mantids form a well-established lineage of insects, the *Dictyoptera*. In fact, termites are actually social cockroaches [4], being the family *Cryptocercidae* their closest relative and the *Mantodea* (mantids) the sister group to the clade comprising cockroaches and termites [5]. Six families of termites (collectively called lower termites) share with *Cryptocercus* spp. the unusual ability to degrade lignocellulosic plant material, carried out by the metabolic activities of the bacteria and protists of their gut microbiota [6–9]. Higher termites have lost their gut protists, having only bacteria. They are represented by a single highly diversified family, the *Termitidae*.

Modern cockroaches are thought to have radiated at some time between the late Jurassic and early Cretaceous, ~140 million years ago [10]. There are fundamental differences in the diets of termites and cockroaches. While termites feed almost exclusively on lignocellulose in various stages of decay, many cockroaches subsist on a highly variable diet. Examples of xylophagy in cockroaches are *Cryptocercus* spp. (family *Cryptocercidae*) from East Asia and North America, *Panesthia* spp. (subfamily *Panesthiinae*, family *Blaberidae*) in Australia and Asia, and *Parasphaeria boleiriana* (family *Blaberidae*) from Brazil [11].

In this study, we compared the bacterial gut microbiota of two xylophagous cockroaches, *Cryptocercus puctulatus* and *Parasphaeria boleiriana*. Members of the genus *Cryptocercus* are subsocial cockroaches that inhabit temperate forests of the northern hemisphere, living in extensive galleries excavated within decomposing logs. At present, nine species in the genus are recognized worldwide: two in eastern Eurasia, two in southwestern China, and five in the USA. The distribution of *C*. *punctulatus* extends throughout western Virginia and Pennsylvania. The ecological niche for the five Nearctic *Cryptocercus* species lies within a small range of the spectrum of annual mean temperatures and precipitation that characterize this region: 6–17°C and 140–470 mm/m^2^, respectively [12].

*Parasphaeria boleiriana* lives in the remnants of the semi-deciduous table land of the Atlantic forest in the state of Espirito Santo, Brazil. The genus was previously known for the species *P*. *ovata*, from Chile and Argentina. *P*. *boleiriana* feeds on the softwood of boleira (the tree *Joannessia princeps*). It differs from *Cryptocercus* in that it develops and reproduces in a very short time, 2–3 years, rather than >5 years, and survives as an adult for only one season, rather than several years. Brood care by *P*. *boleiriana* is also very short, with a mean of 12 days, compared to several years by *Cryptocercus* [11,13,14].

In lower termites and *Cryptocercus* cockroaches, wood is efficiently digested by their flagellate symbionts (eukaryotes), whereas in higher termites lignocellulosic material (wood, detritus, humus, etc.) is digested by a diverse assemblage of cellulolytic prokaryotes. The clade *Cryptocercus*–termites clearly shows the coevolution of the host with a stable intestinal microbiota essential to its survival [15–17]. By contrast, detailed information on the gut bacterial diversity in *Parasphaeria* (which is phylogenetically distinct from the clade *Cryptocercus*–termites) that allows lignocellulose digestion is still lacking. In the present work, the diversity of metagenomic gut communities of *C*. *punctulatus* and *P*. *boleiriana* was investigated to identify possible shifts in symbiont allegiances during cockroach evolution. We also compared several bacterial phyla associated with the microbiota of cockroaches with other metagenomes databases that correspond on *Cryptocercus*, termites and other insect groups.

## Materials and Methods

### Cockroaches and isolation of bacterial DNA

*Cryptocercus punctulatus* was collected from Virginia (USA) by Dr. Michael Dolan (University of Massachusetts at Amherst, MA, USA), and *Parasphaeria boleiriana* from Brazil by Dr. Philippe Grandcolas (CNRS-National Museum of Natural History, Paris, France). Two individuals of each species were sent to our laboratory in Barcelona, Spain. During transport, the cockroaches were maintained in tubes with wood at room temperature. Immediately after their arrival in the laboratory, they were dissected to extract the whole gut. Cockroaches were dissected with a sterile scalpel. The abdomens of the insects were incised to remove the dorsal cuticle, the gut was collected under sterile de-ionized water, and the hindgut region was separated and placed into an Eppendorf tube for DNA extraction. The hindgut of the insect was homogenized using a FastPrep system (MP Biomedicals Europe) with 0.1-mm glass beads. Bulk DNA was extracted by several washings with phenol-chloroform [18]. All material and solutions used were sterile. Disinfection and dissection were performed in a laminar flow cabinet. During extraction, we worked in an aseptic environment under laminar hood to avoid contamination [19].

### Amplicon library preparation

Amplification of the variable region V1–V2 of the bacterial 16S rDNA gene was utilized to assess gut microbial diversity. Primers used were 8F-338R (5′-AGAGTTTGATCCTGGCTCAG-3′ and 5′-TGCTGCCTCCCGTAGGAGT-3′) for multiplex Roche 454 GS FLX pyrosequencing. Primer design was carried out according to the manufacturer’s instructions. Initial PCR from each DNA was performed four times [20,21]. After PCR, the resulting product was checked for size and purity on an agarose-Sybr safe DNA gel stain (Invitrogen, San Diego, CA, USA). The amplicons were purified using a Pure Link kit (Invitrogen, San Diego, CA, USA) and quantified using Qubit and Bioanalyzer. The pool of amplicons were mixed equimolar (four amplicons for cockroach specie) and then prepared for 454-pyrosequencing according to the manufacturer. Cycling conditions were 94°C for 3 min, followed by 30 cycles of 94°C for 30 s, 56°C for 40 s, 68°C for 40 s, and a final extension step at 68°C for 6 min.

### Bioinformatic analyses

Raw data of both cockroach metagenomes obtained were 5188 and 5788, *Cryptocercus* and *Parasphaeria*, respectively. Data were preprocessed for demultiplex and quality control using a pipeline implemented in GPRO version 1.1 [22]. This pipeline combines the tools Cutadapt [23], Prinseq-Lite [24], and FastQC [25]. Reads less than 250 nucleotides in size and redundant sequences were removed from each metagenome dataset using GPRO and Mothur1.31.2 [26]. This approach resulted in a non-redundant database of 3519 sequences from the *Cryptocercus* dataset and 2744 sequences from the *Parasphaeria* dataset. Data deposition: Bioproject PRJNA284583.

A multiple alignment was constructed for each dataset using the secondary-structure aware infernal aligner [27] combined with Genedoc [28] for manual refinement. Sequences not fulfilling at least 80% of the common core and gaps and non-informative traits were filtered from each alignment by combining the “unique.seqs,” “screen.seqs,” and “filter.seqs” commands of Mothur. CD-HIT-EST from the CD-HIT 4.5.4 package [29] was subsequently used to define clusters of clones within each metagenome with a distance threshold of 0.03 (resulting in a cut-off at the species level). The 3.69 Phylip Dnadist tool [http://evolution.gs.washington.edu/phylip.html] was used to obtain the neighbor-joining (NJ) distance matrix for each alignment. Both matrices were subsequently used to obtain rarefaction curves at different distances (0.03, 0.05, 0.10, and 0.15) and several diversity indices using Mothur1.36.1. Taxonomy was assigned by the Silva database [http://www.arb-silva.de] [30]. Community comparison of both metagenome *Cryptocercus* and *Parasphaeria* was evaluated using the UniFrac Server [31].

### Statistical analyses

Relative abundance of class-phyla data were analyzed using the statistical program R v3.1.3. Kolmogorov-Smirnov test, a nonparametric test, were used to determine statistically significant difference between two samples based on a confidence level of 95.0% (*P* < 0.05 was considered statically significant). A principal components analysis (PCA) was performed to describe the relative abundance at Family level for the *Cryptocercus* (this work plus other *Cryptocercus* metagenomes published in the data base; see results) and *Parasphaeria* cockroaches. PCA data were treated with the pairwise and standardized options. Two components were extracted, and they accounted for 78.86% of the variability in the data.

## Results

### Gut bacterial community in *Cryptocercus* and *Parasphaeria*

After quality control filtering (see Material and Methods), 3519 and 2744 pyrosequencing reads were obtained from the *Cryptocercus* and *Parasphaeria* hindgut, respectively. Operational taxonomic units (OTUs) were defined for multiple cutoffs up to the distance threshold (0.03, 0.05, and 0.1). Rarefaction curves allowed the calculation of OTU richness for both the *Cryptocercus* and the *Parasphaeria* hindgut.

**Fig 1:**
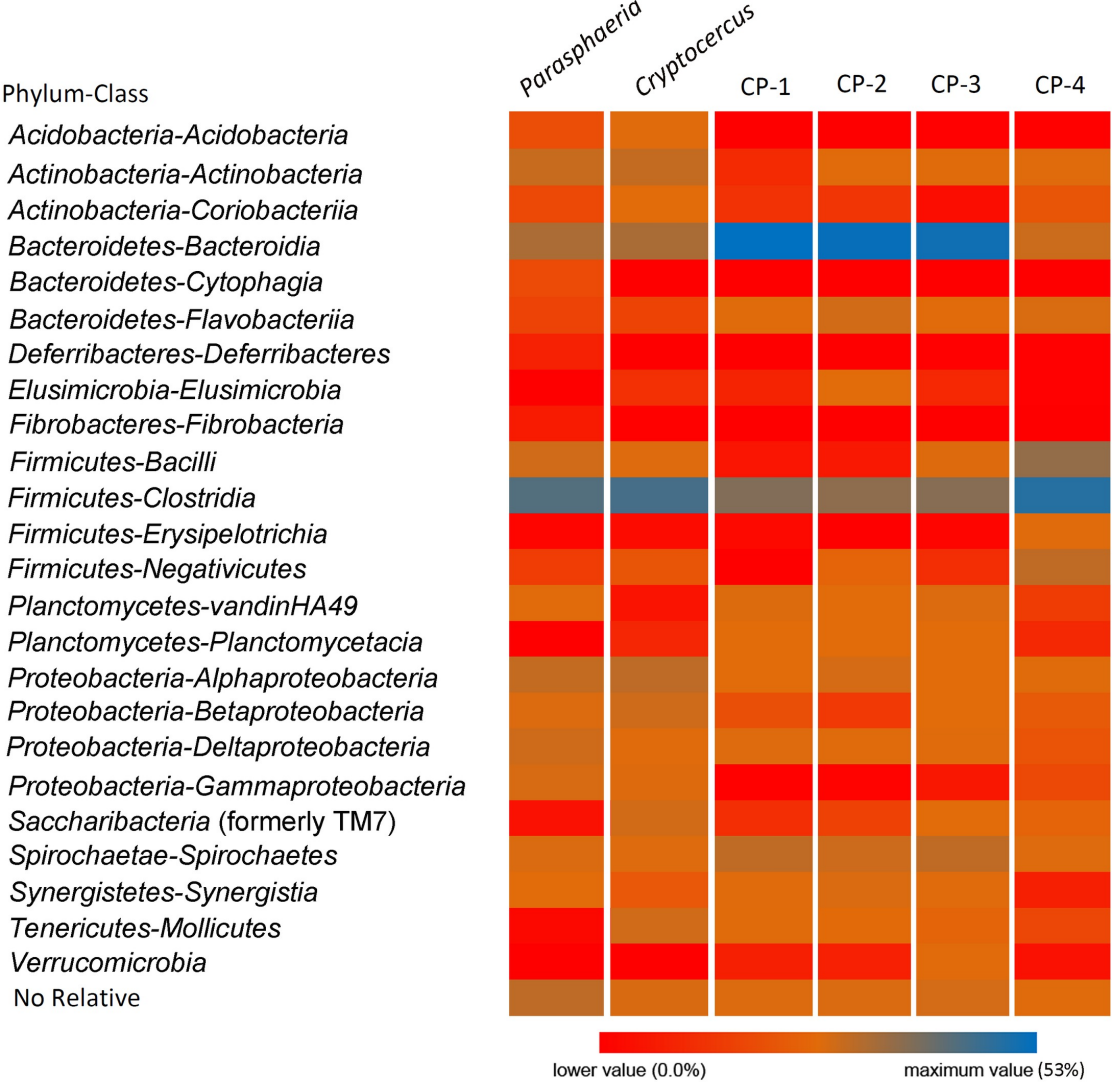
Heatmap of relative abundance of phyla-class bacterial composition of the hindguts of the xylophagous cockroaches *Cryptocercus* and *Parasphaeria*. *Cryptocercus* cockroaches: *Cryptocercus* (this work), CP-1, CP-2, CP-3 (Bioproject PRJNA238270) and CP-4 (PRJNA217467); and *Parasphaeria* (this work).

The calculated rarefaction curves estimated for the two cockroaches showed that the sampling reached an asymptote at the 0.10% genetic distance level (approximately at the family level), indicating that a reasonable number of OTUs had been acquired and that more intensive sampling would likely yield only a few additional OTUs [32,33]. However, rarefaction analysis at either the genus (0.05 distance threshold) or the species (0.03) level indicated that the number of reads analyzed was not sufficient to describe bacterial diversity within the cockroach gut. A total of 1150 and 889 SSU reference OTUs for *Cryptocercus* and *Parasphaeria* respectively were obtained. The representative reads (the longest read of each OUT defined at 97% sequence similarity) are compared to the SILVA reference datasets of the small- (16S/18S) subunit rDNA. The most abundant bacterial phyla in both cockroaches were *Firmicutes*, *Bacteroidetes*, *Proteobacteria*, and *Actinobacteria*. The phylum *Firmicutes* was dominated by members of the *Clostridia* class, and *Bacteroidetes* mostly by members of the class *Bacteroidia*. The class profiles of *Proteobacteria* differed between *Cryptocercus* and *Parasphaeria*. In *Cryptocercus*, *Alphaproteobacteria* and *Betaproteobacteria* dominated whereas in *Parasphaeria*, members of the *Alphaproteobacteria* were the most abundant, followed by *Deltaproteobacteria*, and *Gammaproteobacteria* (Fig 1). The phylum *candidatus* Saccharibacteria (formerly known as *Candidatus* Division TM7) were present in *Cryptocercus* (4% of total bacterial population) but not in *Parasphaeria*. In *Cryptocercus* and *Parasphaeria*, spirochetes represented 1–2% of the total bacterial population. *Elusimicrobia* phyla (formerly Termite Group 1) represented less than 1% of the total bacterial population in *Cryptocercus* but they were not detected in *Parasphaeria*. On the other hand, *Parasphaeria* contained *Deferribacteres* and *Fibrobacteres* that represented less than 0.5% relative abundance *Bacteria*. Relative abundance class-taxon from *Crytocercus* was compared with relative abundance class-taxon from other *Cryptocercus* metagenomes deposited on the NCBI base data. A total of 5 different populations of *Cryptocercus* were analyzed (*Cryptocercus* this work; CP-1, CP-2, CP-3 from BioProject accession number PRJNA238270 [17]; and CP-4 from BioProject PRJNA217467) [34] (Fig 1). Relative abundance and population distribution were different among *Cryptocercus* indicating the “individual” variation of their microbiota, but Kolmogorov-Smirnov test showed that there were not significant differences at 95% confidence level (*P* ≥ 0.05) between *Cryptocercus* and CP-1; *Cryptocercus* and CP-2; *Cryptocercus* and CP-3 and *Cryptocercus* and CP-4. But there were significant differences (*P* < 0.05) between *Cryptocercus* and *Parasphaeria* (similar result was obtained using the unweighted UniFrac analysis, see below). PCA analysis revealed similarities among the bacterial microbiota of the different *Cryptocercus* metagenomes analyzed (S1 Fig). In this case, we considered “individual cockroach” as an individual member that probably represented the gut microbiota from a “like-colony” (several individuals living together), because cockroaches can be considered a gregarious insect [35,36].

**Fig 2:**
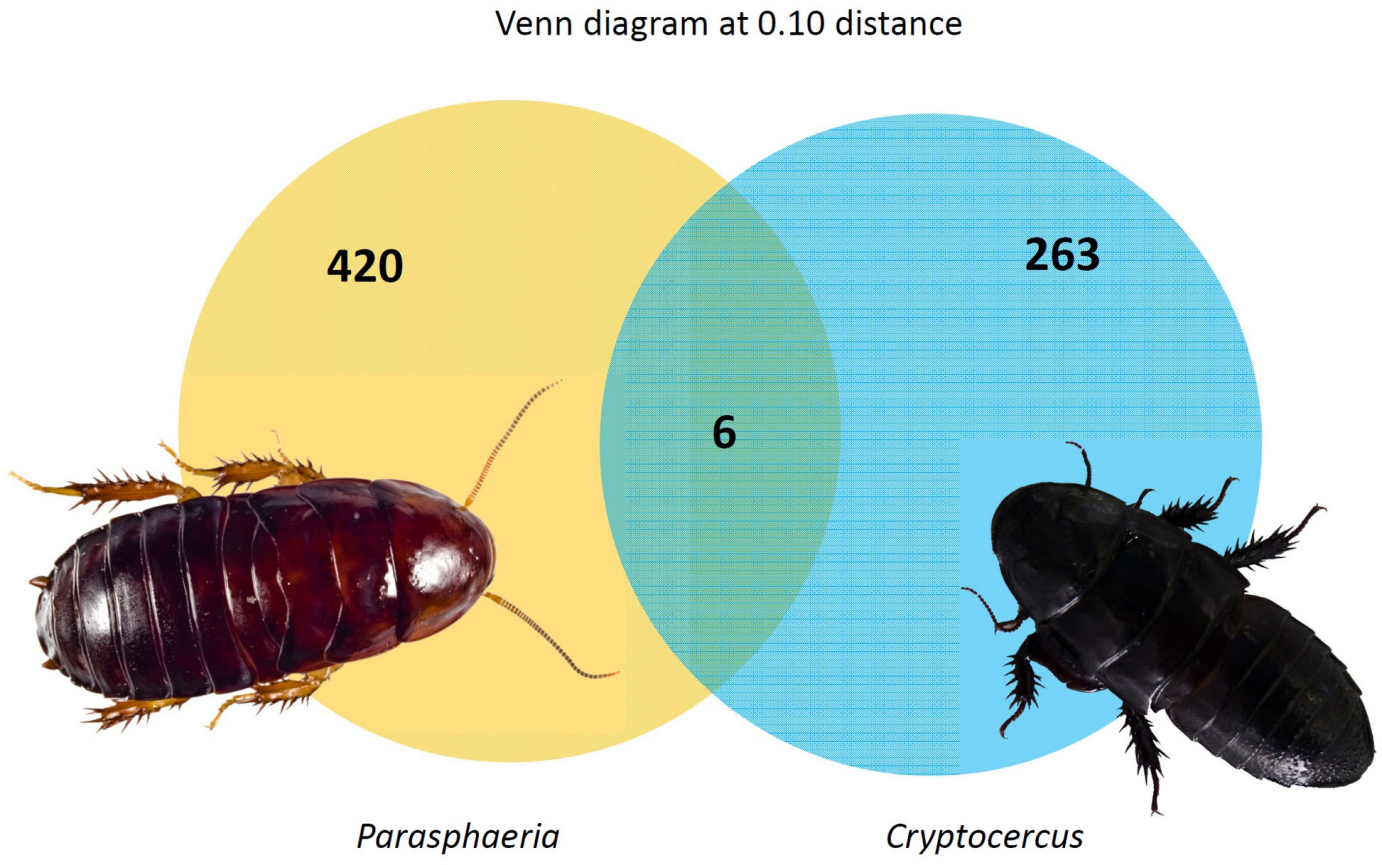
Venn diagram. *Parasphaeria* and *Cryptocercus* shared 6 OUT at 0.10 distances. Figure showed the two cockroaches. (Photo by M. Berlanga and R. Duro).

At 0.03 distances, Shannon’s diversity index showed that the intestinal tracts of *Cryptocercus* and *Parasphaeria*, 5.6 and 5.89 respectively, support a higher diversity community of bacteria similar to other wood or herbivorous-feeding insects [34,37] Bacterial community similarities between *Cryptocercus* and *Parasphaeria* were quantified based on phylogenetic relatedness by unweighted UniFrac. The analyses indicated that the composition of the bacterial communities from the two cockroaches differed significantly (*P* < 0.03). But, the Venn diagram generated by Mothur v1.36.1 from *Cryptocercus* and *Parasphaeria* at a genetic distance of 0.10 showed that six OTUs were shared and were related phylogenetically one to the phyla *Spirochaetes* (related to *Treponema* cluster I), two *Bacteroidetes* (Family *Porphyromonadaceae* belonging to *Dysgonomonas* and *Parabacteroides* genera) and tree *Firmicutes* (class *Clostridia*, uncultured bacteria of the Family XIII) (Fig 2). S1 and S2 Tables indicated the different OTUs and their taxonomic identification at genus level from *Cryptocercus* and *Parasphaeria* metagenomes, respectively.

### OTUs assigned to *Spirochaetes*

**Fig 3:**
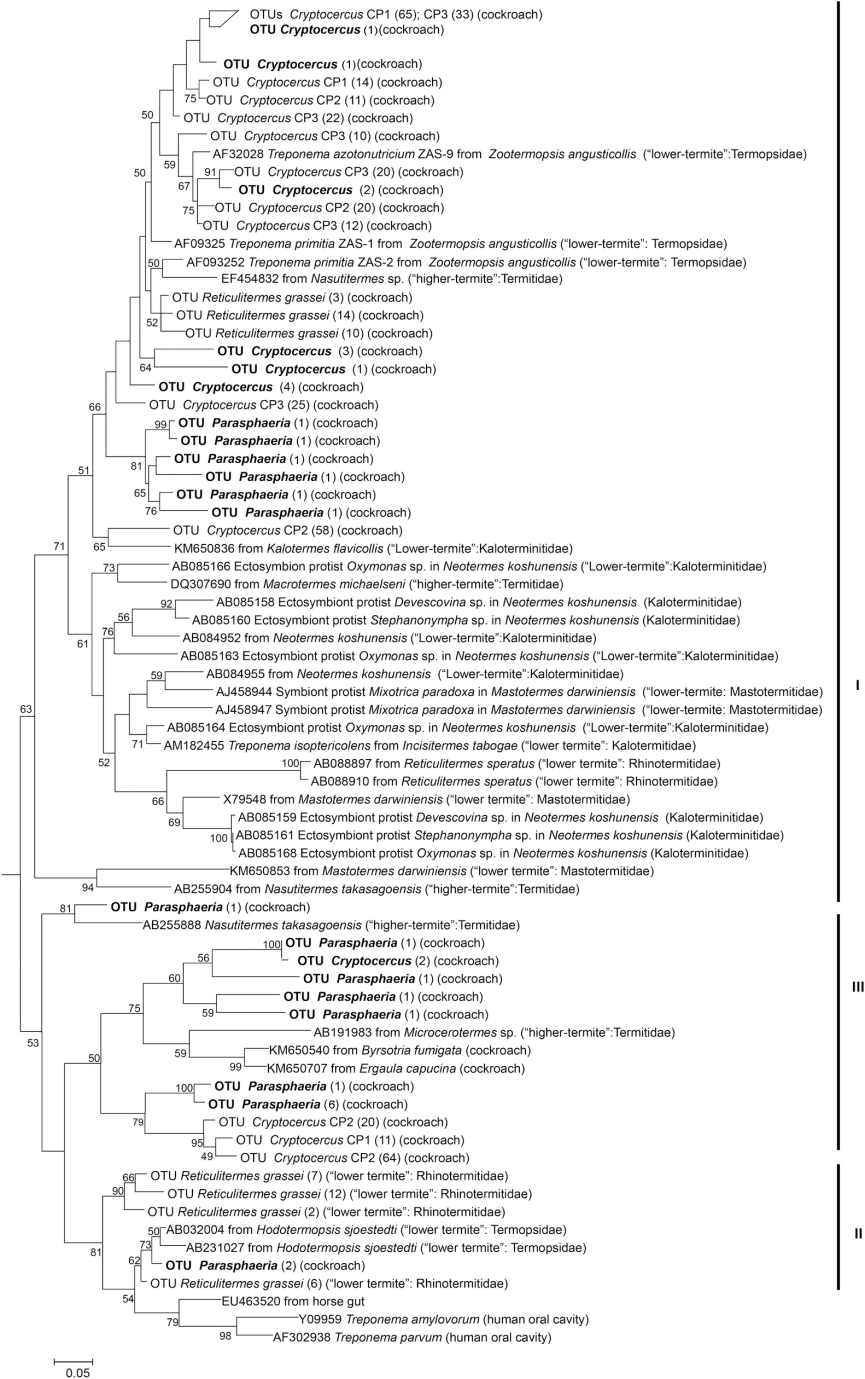
Phylogenetic tree based on maximum-parsimony (MP) and maximum-likelihood (ML) analyses depicting the relationship among the pyrotags affiliated with the *Treponema* I, II, and III clades in termites and cockroaches. Reference OTU from *Cryptocercus* and *Parasphaeria* were in bold. Other references OTU were obtained from *Cryptocercus* CP-1, CP-2 and CP-3 (Bioproject PRJNA238270) and *Reticulitermes grassei* [68]. In parentheses, the number of OTUs found repeatedly (at 0.05% genetic distance level). One thousand bootstrap trees were generated; bootstrap confidence levels, as percentages (only values >50%), are shown at tree nodes.

Spirochetal OTUs from *Cryptocercus* and *Parasphaeria* fell into three clusters, designated *Treponema*-termite clusters I, II, and III (Fig 3). *Treponema*-termite cluster I comprises both ectosymbionts attached to protists and free-swimming gut spirochetes from lower and higher termites. OTUs from *Cryptocercus* and *Parasphaeria* were grouped with free-swimming *Treponema* based on their affiliation with sequences of the isolates *Treponema primitia* or *T*. *azotonutricium* and other sequences of *Treponema* from higher termites (Fig 3). Sequences obtained from other metagenomes of *Cryptocercus* (CP1; CP2; CP3) Bioproject PRJNA238270 also cluster with pyrotags detected in this work. The second cluster, *Treponema-*termite cluster II, is much smaller than *Treponema*-termite cluster I and also much less diverse. It contains only sequences from lower termites and generally they were described in *Reticulitermes* and *Hodotermopsis* termites [38,39]. One OTU from *Parasphaeria* grouped with several sequences previously reported as belonging to *Treponema*-termite cluster II. Several OTUs from *Cryptocercus* and *Parasphaeria* grouped with *Treponema*-termite cluster III that contained *Treponema* sequences from other cockroaches and from higher termites (Fig 2). Data suggested that *Spirochaetes* from *Cryptocercus* and *Parasphaeria* could be free living bacteria present in the cockroaches before acquisition of flagellates’ protists by *Cryptocercus* cockroaches. *Treponema* detected in *Cryptocercus* and *Parasphaeria* were different to other *Treponema* described in other habitats such as human oral cavity (Fig 3).

### OTUs assigned to *Bacteroidetes*

**Fig 4:**
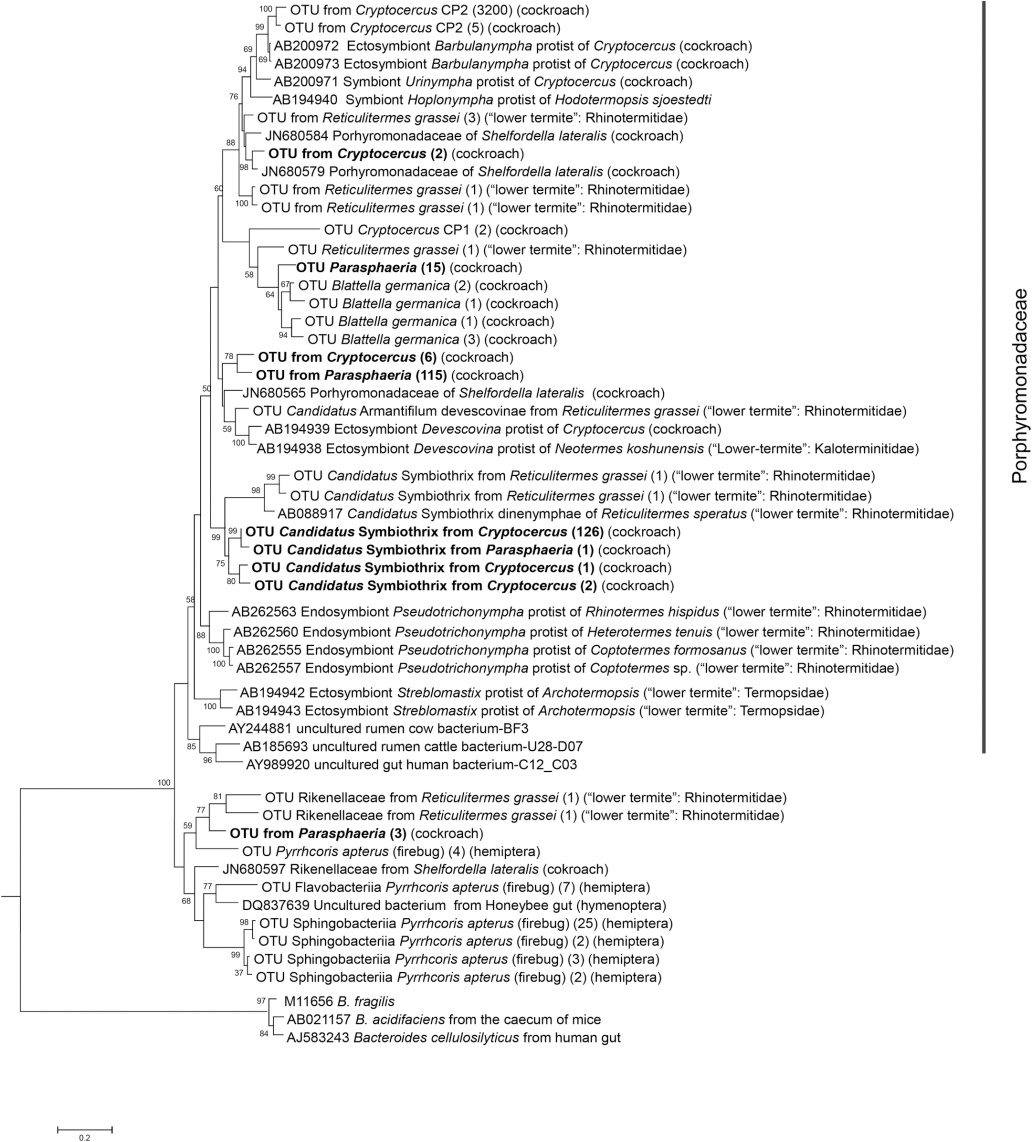
Phylogenetic tree based on maximum-parsimony (MP) and maximum-likelihood (ML) analyses depicting the relationship among the pyrotags affiliated with *Bacteroidetes*. Reference OTU from *Cryptocercus* and *Parasphaeria* were in bold. Other references OTU were obtained from *Cryptocercus* CP-1, CP-2 and CP-3 (Bioproject PRJNA238270), *Reticulitermes grassei* [68], *Blattella germanica* (Bioproject PRJEB3414) and *Pyrrhcoris apterus* (firebug) Bioproject PRJNA171139. In parentheses, the number of OTUs found repeatedly (at 0.10% genetic distance level). One thousand bootstrap trees were generated; bootstrap confidence levels, as percentages (only values >50%), are shown at tree nodes.

In *Cryptocercus* and *Parasphaeria*, the dominant class was *Bacteroidia* and the family *Porphyromonadaceae*. OTUs from *Cryptocercus* were related to *Bacteroidetes* previously described sequences from different species of the protists *Barbulanympha*, *Urinympha*, *Hoplonympha*, and *Mixotricha*. Both *Barbulanympha* and *Urinympha* occur exclusively in the gut of *Cryptocercus* [40]. Several OTUs reference sequences from different cockroaches and termites were closely related to the before mentioned cluster. OTUs from *Crytocercus* also clustered with ectosymbiont *Candidatus* Symbiotrix of the protist *Dinenympha*. No representatives OTUs from *Cryptocercus* or *Parasphaeria* were clustered with other sequences belonging to termite symbionts protists *Pseudotrichonympha* or *Streblomastix* (Fig 4). *Candidatus* Symbiothrix, *Dysgonomonas*, *Parabacteroides*, *Paludibacter* and *Tannerella* were the major genera detected based on the Silva database. *Porphyromonadaceae* detected in cockroaches and termites were phylogenetically different from others obtained from cattle intestinal tracts (Fig 4).

### Clones assigned to *Deltaproteobacteria*

**Fig 5:**
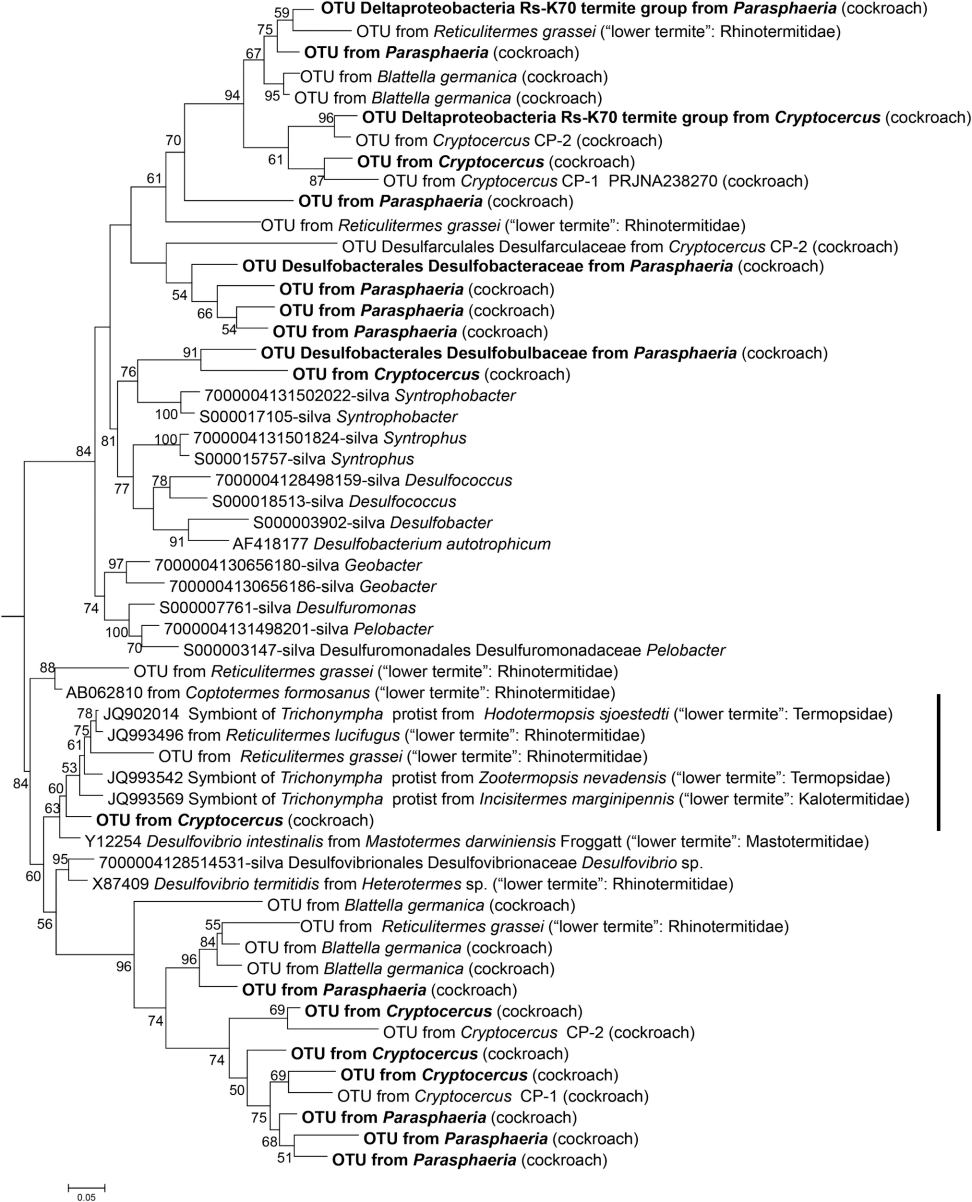
Phylogenetic tree based on maximum-parsimony (MP) and maximum-likelihood (ML) analyses depicting the relationship among the pyrotags affiliated with *Deltaproteobacteria*. Reference OTU from *Cryptocercus* and *Parasphaeria* were in bold. Other references OTU were obtained from *Cryptocercus* CP-2 (Bioproject PRJNA238270), *Reticulitermes grassei* [68], *Blattella germanica* (Bioproject PRJEB3414) and *Pyrrhcoris apterus* (firebug) (Bioproject PRJNA171139). In parentheses, the number of OTUs found repeatedly (at 0.05% genetic distance level). One thousand bootstrap trees were generated; bootstrap confidence levels, as percentages (only values >50%), are shown at tree nodes.

The phylum *Proteobacteria* represented 17.3 and 19.5 of relative pyrotags sequences obtained in *Cryptocercus* and *Parasphaeria*, respectively. *Deltaproteobacteria* represented 1.7 and 5.8 of the *Proteobacteria* sequences in *Cryptocercus* and *Parasphaeria*, respectively. The detected *Deltaproteobacteria* OTUs clustered with the families *Desulfobacteraceae* and *Desulfovibrionaceae* families. *Desulfobacteraceae* could be grouped in two clusters: one, grouped sequences obtained from cockroaches (*Cryptocercus*, *Parasphaeria* and *Blattella germanica*) and *Reticulitermes* termite; and second, OTUs detected from *Cryptocercus* and *Parasphaeria* clustered with syntrophic *Deltaproteobacteria*, such as *Syntrophobacter* spp. (Fig 5) OTUs from *Cryptocercus* belonging to the family *Desulfovibrionaceace* clustered with other sequences described from the termite gut related with the protist *Trichonympha*. In the *Desulfovibrionaceae* family, other group that contains sequences from several cockroaches and termites but were not related to symbionts of protists could be observed (Fig 5).

## Discussion

Insects contribute to an enormous diversity of symbiotic relationships. Microbial symbiosis probably played a central role in the evolutionary success of these organisms, allowing their adaptation to ecological niches that are nutritionally deprived and/or unbalanced (e.g., wood, plant sap or blood). The hindgut of insects is persistently colonized by opportunistic and commensal microbiota largely structured by exogenous (diet and local environment) and endogenous (gut environment) factors [17,21,41]. Transient bacteria acquired from the food and environment sources may complicate the apparent composition of gut microbial communities, but dynamic core gut microbiota (commensal) have been maintained even after environmental shifts [42–45]. Some termite gut-specific bacterial lineages have been observed that were not detected on environmental soil [45]. In this work we studied specific bacterial groups (phyla such as *Spirochaetes*, *Bacteroidetes* and *Deltaproteobacteria*) that have been described as a characteristic microbiota in the gut of termites and *Cryptocercus* [34,41].

Comparison of the relative abundance of class taxa among different *Cryptocercus* metagenomes (our work and four *Cryptocercus* from the Bioprojects PRJNA238270 [17] and PRJNA217467 [37]) indicated individual variation of their microbiota, but there were no significant differences at 95% confidence level (*P* ≥ 0.05, Kolmogorov-Smirnov test) (Fig 1). “Individual” microbiota referred to several representatives’ members of a gregarious community of cockroaches living in a particular place, as it has been pointed out that there are few solitary cockroaches [35]. The “social” structure of *Cryptocercus* is the equivalent of a newly founded termite colony. After the eggs have hatched, adults feed the first few instars on hindgut fluids (proctodeal trophallaxis) [35]. The neonatal digestive tract is free of microbes, and the establishment of the full complement of microbial symbionts is a sequential process that varies in length between species. Typically, it is not complete until the third instar, when nutritional independence is possible, although close contact with adults is maintained [46]. Aggregation of the German cockroach, *Blattella germanica*, is regulated by fecal aggregation agents (pheromones), including volatile carboxylic acids [36]. The termite worker caste transfers food stomodeally (by regurgitation) and/or proctodeally (by excretion with the hindgut contents). Both oral trophallaxis (feeding) and coprophagy can promote a secure transmission of commensal microbiota between gregarious cockroaches [47–50] or members of a colony (termites).

In *Dictyoptera*, the transition from an omnivorous to a wood-feeding lifestyle had a strong impact on the microbial community structure, as observed in *Cryptocercus* and (lower) termites which included the acquisition of cellulolytic flagellates [17,41]. The complete loss of all flagellates in higher termites constituted another hallmark in the evolution of *Isoptera* [34,41]. But, other wood-feeding cockroaches, such as *Panesthia* spp., *Salganea* spp. and *Parasphaeria* spp. did not support the characteristic community of gut protists observed in the cockroach *Cryptocercus* [51,52]. In omnivorous cockroaches such as *Periplaneta americana*, their cellulose-rich diets have favored high protist numbers (e.g., *Nyctotherus ovalis*, *Ciliophara*), resulting in high cellulase activity. In fact, *N*. *ovalis* is responsible for most of the cellulolytic activity of *P*. *americana* [53]. The hindguts of the wood-feeding cockroach subfamily *Panesthiinae* harbor ciliated protists but they are probably not associated with the digestion of wood; rather, they are close related to the genus *Nyctotherus*, present in other cockroaches [54,55]. In thegenus *Parasphaeria*, while lacking the specific gut flagellates harbored in lower termites and *Cryptocercus*, many bacterial taxa were found. Of particular interest are *Spirochaetes* (Fig 3), *Bacteroidetes* (Fig 4), and *Deltaproteobacteria* (Fig 5), which are closely related to bacterial symbionts that specifically colonize the surface or interior of termite gut flagellates [6,56,57]. The sequences of *Spirochaetes*, *Bacteroidetes* and *Deltaproteobacteria* detected in *Parasphaeria* and other cockroaches likely represent free-living relatives present in a common ancestor of cockroaches before their association with specific protists [44,57,58].

In *Cryptocercus* and *Parasphaeria*, *Spirochaetes* accounted for 1–2% of the total population. They are rare or not detected in omnivorous cockroaches, such as *Shelfordella*, *Periplaneta*, or in the xylophagous *Panesthia angustipennis* [37,44,54,59,60], but they are abundant in termites, especially in higher termites [61]. Spirochetes specialize in metabolic interactions with their hosts or other co-occurring microorganisms [62]. The main compounds produced by spirochetes are acetate, H_2_, and CO_2_, all of which are consumed by sulfate-reducing bacteria and methanogens (with both groups represented in termites). Acetate produced by the gut microbiota supports up to 100% of the respiratory requirement of termites [63]. Spirochetes from termite hindguts possess homologues of a nitrogenase gene (*nifH*) and exhibit nitrogenase activity [57]. This observation implicates spirochetes in the nitrogen nutrition of termites, whose food is typically low in nitrogen. Spirochete populations can stably maintain the gut habitat by supplying carbon sources and electron donors to other resident microbial populations and to the host.

*Bacteroidetes* OTUs from *Cryptocercus* grouped closely with phylotypes previously described from different species of the protist *Barbulanympha*. The genera *Candidatus* Symbiothrix, *Dysgonomonas*, *Parabacteroides*, *Paludibacter* and *Tannerella* were the major genera identified. In *Parasphaeria*, based on the Silva database, the major genera were *Paludibacter*, *Parabacteroides* and *Candidatus* Symbiothrix. These genera may represent the termite-specific bacterial lineages reported in termites [64,65]. Members of *Bacteroidetes* are thought to be specialized in the degradation of complex organic matter, including lignocellulosic compounds [66]. *Bacteroidetes* were also related to diazotrophic bacteria such as *Azobacteroides pseudotrichonympha* that provide amino acids and cofactors for the nutrition of the host protist and of the cockroach host [51,57].

*Deltaproteobacteria* OTUs in *Cryptocercus* and *Parasphaeria* hindguts were assigned to the families *Desulfobacteriaceae* and *Desulfovibrionaceae*. Both groups are strict anaerobes that are capable of sulfate-reduction. Sulfate-reducing bacteria (SRB) are crucial to the final step of carbon recycling and to the sulfur cycle in anaerobic ecosystems [67]. In addition to being important hydrogenotrophs (H_2_-consuming microorganisms), SRB contribute to the anoxic milieu of the gut by producing hydrogen sulfide and by removing oxygen together with hydrogen or low molecular weight organic or reduced sulfur compounds. H_2_ fluxes almost certainly play a significant role in shaping community structure, as H_2_ is both widely utilized as a microbial substrate and strongly influences the thermodynamics of the reactions in which it participates [67].

The molecular characterization carried out in this work revealed that bacterial community differ significantly between *Cryptocercus* and *Parasphaeria*; but they shared several bacterial genera found in other termites and cockroaches. Of special interest were several common OTUs detected in the intestinal tract belonging to *Spirochaetes*, *Bacteroidetes* and *Firmicutes* (*Clostridia*) that could represent a core microbiota essential to hydrolyze plant compounds and to provide nitrogen sources such as amino acids to their host (Fig 2). Wood-feeding *Cryptocercus* and *Parasphaeria* were phylogenetically distant cockroaches, but several bacterial groups were present in both cockroaches and were shared also with termites. Those bacteria may derive from the microbiota of a common ancestor before the diversification of cockroaches, subsequently diversified and adapted in each host. The microbiota depends on the hosts’ feeding behavior and secretions. The composition and physical form of the food changes as it passes down the gastrointestinal tract, offering microbes at different locations a changing complement of nutrients. Finally, the host obtains multiple nutrients in appropriate quantities and balance to optimally perform their biological function.

## Supporting Information

S1 Fig Principal Component Analysis (PCA) for *Cryptocercus* and *Parasphaeria* cockroaches based on relative abundance of bacterial taxa determined with Silva base data. *Cryptocercus* cockroaches: *Cryptocercus* (this work), CP-1, CP-2, CP-3 (Bioproject PRJNA238270) and CP-4 (PRJNA217467); *Parasphaeria* (this work); *Apis mellifera* (PRJNA82239) as an out group of the *Dictyoptera* insect.
(TIF)

S1 Table Representative OTUs at 0.05 distance for *Cryptocercus*. (PDF)

S2 Table Representative OTUs at 0.05 distance for *Parasphaeria*. (PDF)
