# Supplementary figures and images for: Gut Bacterial Community of the Xylophagous Cockroaches Cryptocercus punctulatus and Parasphaeria boleiriana
Source: PLoS One. 2016 Apr 7;11(4):e0152400. doi: 10.1371/journal.pone.0152400 (PMC4824515; doi:10.1371/journal.pone.0152400)

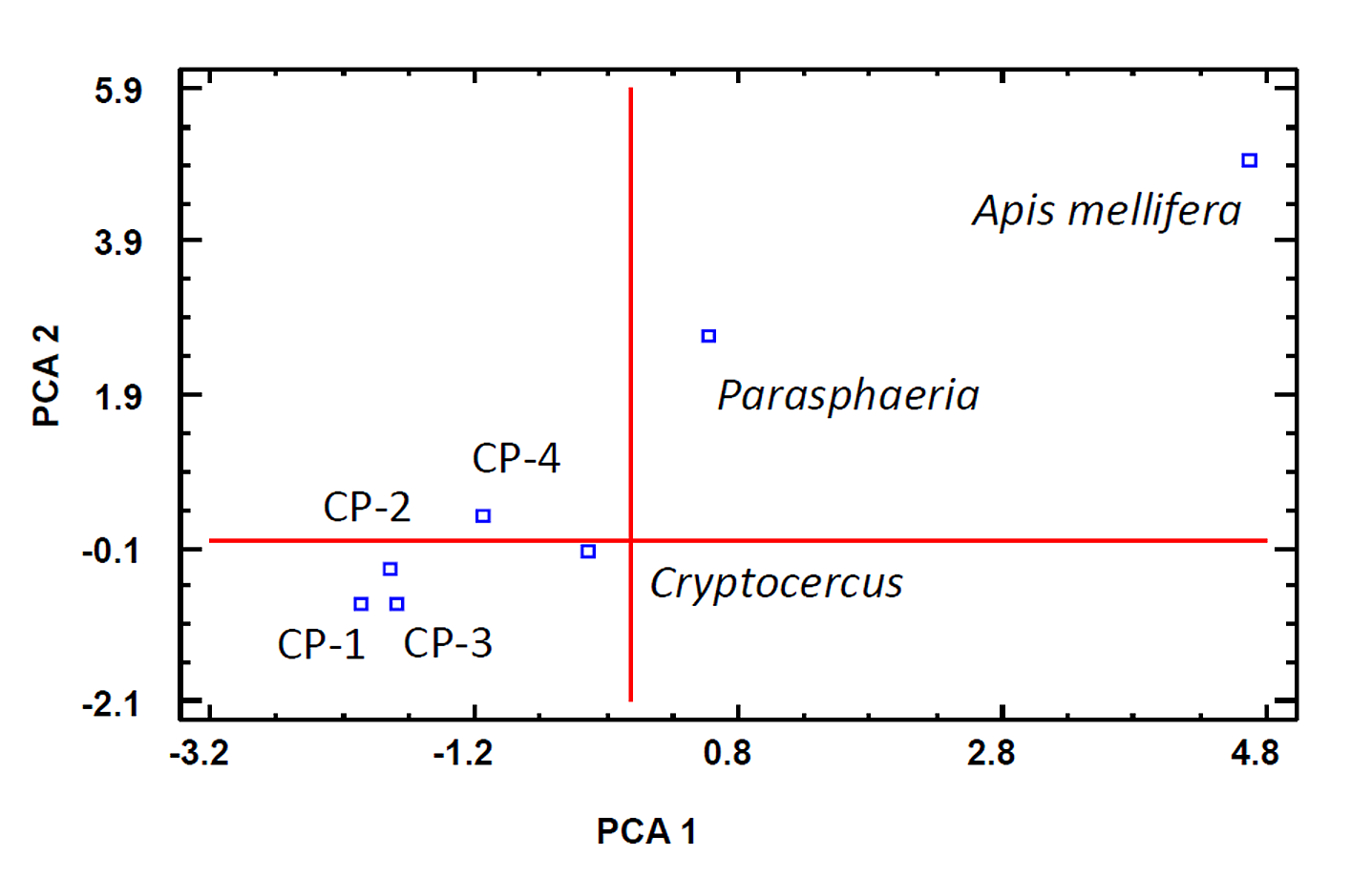

Supplement: S1 Fig — Cryptocercus cockroaches: Cryptocercus (this work), CP-1, CP-2, CP-3 (Bioproject PRJNA238270) and CP-4 (PRJNA217467); Parasphaeria (this work); Apis mellifera (PRJNA82239) as an out group of the Dictyoptera insect. (TIF) [file pone.0152400.s001.tif]
