# Supplementary material for: Gut Bacterial Community of the Xylophagous Cockroaches Cryptocercus punctulatus and Parasphaeria boleiriana
Source: PLoS One. 2016 Apr 7;11(4):e0152400. doi: 10.1371/journal.pone.0152400 (PMC4824515; doi:10.1371/journal.pone.0152400)
Supplement: S1 Table — (PDF) [file pone.0152400.s002.pdf]

S1 Table. Representative OTU at 0.05 distance for *Cryptocercus*.

Classification	Representative_Seq.	OTUs
Bacteria Acidobacteria Acidobacteria Acidobacteriales Acidobacteriaceae Granulicella	HUT5UCF08JMN9E	1
Bacteria Acidobacteria Acidobacteria Acidobacteriales Acidobacteriaceae	HUT5UCF08I4M3S	2
Bacteria Acidobacteria Acidobacteria Acidobacteriales Acidobacteriaceae	HUT5UCF08JDYI6	1
Bacteria Acidobacteria Acidobacteria Acidobacteriales Acidobacteriaceae	HUT5UCF08JN4RG	3
Bacteria Acidobacteria Acidobacteria Acidobacteriales Acidobacteriaceae Uelmatobacter	HUT5UCF08I3BYZ	1
Bacteria Acidobacteria Acidobacteria Acidobacteriales Acidobacteriaceae Acidobacterium	HUT5UCF08JQI4K	1
Bacteria Acidobacteria Acidobacteria Acidobacteriales Acidobacteriaceae Acidobacterium	HUT5UCF08JQOZ3	3
Bacteria Acidobacteria Acidobacteria Acidobacteriales Acidobacteriaceae Acidobacterium	HUT5UCF08JTRKG	1
Bacteria Acidobacteria Acidobacteria Acidobacteriales Acidobacteriaceae Acidobacterium	HUT5UCF08I45VN	6
Bacteria Acidobacteria Acidobacteria Acidobacteriales Acidobacteriaceae Acidobacterium	HUT5UCF08I8RT5	2
Bacteria Acidobacteria Acidobacteria Acidobacteriales Acidobacteriaceae Acidobacterium	HUT5UCF08JFHV3	1
Bacteria Acidobacteria Acidobacteria Acidobacteriales Acidobacteriaceae Acidobacterium	HUT5UCF08JF0JO	1
Bacteria Acidobacteria Acidobacteria Acidobacteriales Acidobacteriaceae Acidobacterium	HUT5UCF08JVLWQ	1
Bacteria Acidobacteria Acidobacteria Acidobacteriales Acidobacteriaceae Acidobacterium	HUT5UCF08I6VCB	1
Bacteria Acidobacteria Acidobacteria Acidobacteriales Acidobacteriaceae Acidobacterium	HUT5UCF08I213Z	1
Bacteria Acidobacteria Acidobacteria Acidobacteriales Acidobacteriaceae Acidobacterium	HUT5UCF08JE7BF	1
Bacteria Acidobacteria Acidobacteria Acidobacteriales Acidobacteriaceae Edaphobacter	HUT5UCF08JC0V5	3
Bacteria Acidobacteria Acidobacteria Acidobacteriales Acidobacteriaceae Edaphobacter	HUT5UCF08JN0SK	1
Bacteria Acidobacteria Acidobacteria Acidobacteriales Acidobacteriaceae Granulicella	HUT5UCF08I6AFW	1
Bacteria Acidobacteria Acidobacteria Acidobacteriales Acidobacteriaceae Granulicella	HUT5UCF08JE8WQ	2
Bacteria Acidobacteria Acidobacteria Acidobacteriales Acidobacteriaceae Telmatobacter	HUT5UCF08I2Q9L	5
Bacteria Acidobacteria Acidobacteria Acidobacteriales Acidobacteriaceae Telmatobacter	HUT5UCF08JQIK	3
Bacteria Actinobacteria Acidimicrobiia Acidimicrobiales Acidimicrobiaceae	HUT5UCF08I25RH	1
Bacteria Actinobacteria Actinobacteria Corynebacteriales Corynebacteriaceae Corynebacterium	HUT5UCF08I745S	1
Bacteria Actinobacteria Actinobacteria Corynebacteriales Mycobacteriaceae Mycobacterium	HUT5UCF08JQZMZ	1
Bacteria Actinobacteria Actinobacteria Frankiales Acidothermaceae Acidothermus	HUT5UCF08I7IX9	1
Bacteria Actinobacteria Actinobacteria Frankiales Sporichthyaceae Sporichthya	HUT5UCF08JPBA0	1
Bacteria Actinobacteria Actinobacteria Micrococcales Dermabacteraceae Devriesea	HUT5UCF08JPVBX	1
Bacteria Actinobacteria Actinobacteria Micrococcales Dermacoccaceae Kytococcus	HUT5UCF08JOJ1Y	1

Bacteria Actinobacteria Actinobacteria Micrococcales Intrasporangiaceae Ornithinicoccus	HUT5UCF08I4I6T	1
Bacteria Actinobacteria Actinobacteria Micrococcales Microbacteriaceae Agromyces	HUT5UCF08I9C0W	2
Bacteria Actinobacteria Actinobacteria Micrococcales Microbacteriaceae Agromyces	HUT5UCF08JCYO0	1
Bacteria Actinobacteria Actinobacteria Micrococcales Microbacteriaceae Agromyces	HUT5UCF08JPNC2	3
Bacteria Actinobacteria Actinobacteria Micrococcales Microbacteriaceae Agromyces	HUT5UCF08I2FFT	2
Bacteria Actinobacteria Actinobacteria Micrococcales Microbacteriaceae Gryllotalpicola	HUT5UCF08I7DJ8	1
Bacteria Actinobacteria Actinobacteria Micrococcales Microbacteriaceae Lysinimonas	HUT5UCF08JKDPT	1
Bacteria Actinobacteria Actinobacteria Micrococcales Microbacteriaceae Microbacterium	HUT5UCF08JTTYE	1
Bacteria Actinobacteria Actinobacteria Micrococcales Microbacteriaceae Salinibacterium	HUT5UCF08JI0BA	4
Bacteria Actinobacteria Actinobacteria Propionibacteriales Nocardioidaceae Nocardioides	HUT5UCF08JFT6D	3
Bacteria Actinobacteria Actinobacteria Propionibacteriales Nocardioidaceae Nocardioides	HUT5UCF08I3BAL	2
Bacteria Actinobacteria Actinobacteria Propionibacteriales Propionibacteriaceae uncultured	HUT5UCF08I35OR	4
Bacteria Actinobacteria Actinobacteria Propionibacteriales Propionibacteriaceae uncultured	HUT5UCF08JI8SG	1
Bacteria Actinobacteria Actinobacteria Propionibacteriales Propionibacteriaceae uncultured	HUT5UCF08JJG0F	1
Bacteria Actinobacteria Actinobacteria Propionibacteriales Propionibacteriaceae uncultured	HUT5UCF08JMH8M	1
Bacteria Actinobacteria Actinobacteria Propionibacteriales Propionibacteriaceae uncultured	HUT5UCF08JN0H8	1
Bacteria Actinobacteria Actinobacteria Propionibacteriales Propionibacteriaceae Aestuariimicrobium	HUT5UCF08I5CCQ	5
Bacteria Actinobacteria Actinobacteria Propionibacteriales Propionibacteriaceae Aestuariimicrobium	HUT5UCF08I5JV3	1
Bacteria Actinobacteria Actinobacteria Propionibacteriales Propionibacteriaceae Aestuariimicrobium	HUT5UCF08I69HZ	1
Bacteria Actinobacteria Actinobacteria Propionibacteriales Propionibacteriaceae Aestuariimicrobium	HUT5UCF08JFV0M	1
Bacteria Actinobacteria Actinobacteria Propionibacteriales Propionibacteriaceae Brooklawnia	HUT5UCF08I697S	1
Bacteria Actinobacteria Actinobacteria Propionibacteriales Propionibacteriaceae Propionibacterium	HUT5UCF08JE73S	1
Bacteria Actinobacteria Actinobacteria Propionibacteriales Propionibacteriaceae Propioniciclava	HUT5UCF08I9Q1S	1
Bacteria Actinobacteria Actinobacteria Propionibacteriales Propionibacteriaceae Propionicimonas	HUT5UCF08JENMN	2
Bacteria Actinobacteria Coriobacteriia Coriobacteriales Coriobacteriaceae uncultured	HUT5UCF08JP07R	2
Bacteria Actinobacteria Coriobacteriia Coriobacteriales Coriobacteriaceae uncultured	HUT5UCF08JQ6J0	1
Bacteria Actinobacteria Coriobacteriia Coriobacteriales Coriobacteriaceae uncultured	HUT5UCF08I2SG6	1
Bacteria Actinobacteria Coriobacteriia Coriobacteriales Coriobacteriaceae uncultured	HUT5UCF08JF4N5	1
Bacteria Actinobacteria Coriobacteriia Coriobacteriales Coriobacteriaceae uncultured	HUT5UCF08I3F3L	2
Bacteria Actinobacteria Coriobacteriia Coriobacteriales Coriobacteriaceae uncultured	HUT5UCF08JKAZ0	1
Bacteria Actinobacteria Coriobacteriia Coriobacteriales Coriobacteriaceae uncultured	HUT5UCF08JNCQ5	1
Bacteria Actinobacteria Coriobacteriia Coriobacteriales Coriobacteriaceae uncultured	HUT5UCF08JLXFY	1
Bacteria Actinobacteria Coriobacteriia Coriobacteriales Coriobacteriaceae uncultured	HUT5UCF08JAT87	1

Bacteria Actinobacteria Coriobacteriia Coriobacteriales Coriobacteriaceae uncultured	HUT5UCF08JGD3K	2
Bacteria Bacteroidetes Bacteroidia Bacteroidales M2PB4-65_termite_group	HUT5UCF08I5GFB	1
Bacteria Bacteroidetes Bacteroidia Bacteroidales Porphyromonadaceae uncultured	HUT5UCF08I375W	5
Bacteria Bacteroidetes Bacteroidia Bacteroidales Porphyromonadaceae uncultured	HUT5UCF08JI1TH	2
Bacteria Bacteroidetes Bacteroidia Bacteroidales Porphyromonadaceae uncultured	HUT5UCF08JRJMB	1
Bacteria Bacteroidetes Bacteroidia Bacteroidales Porphyromonadaceae uncultured	HUT5UCF08JKM9V	3
Bacteria Bacteroidetes Bacteroidia Bacteroidales Porphyromonadaceae Candidatus _Symbiothrix	HUT5UCF08I4P5V	2
Bacteria Bacteroidetes Bacteroidia Bacteroidales Porphyromonadaceae Candidatus _Symbiothrix	HUT5UCF08JCV64	8
Bacteria Bacteroidetes Bacteroidia Bacteroidales Porphyromonadaceae Candidatus _Symbiothrix	HUT5UCF08JHVO6	8
Bacteria Bacteroidetes Bacteroidia Bacteroidales Porphyromonadaceae Candidatus _Symbiothrix	HUT5UCF08JCW8N	6
Bacteria Bacteroidetes Bacteroidia Bacteroidales Porphyromonadaceae Candidatus _Symbiothrix	HUT5UCF08JT9AZ	5
Bacteria Bacteroidetes Bacteroidia Bacteroidales Porphyromonadaceae Candidatus _Symbiothrix	HUT5UCF08JH3KW	1
Bacteria Bacteroidetes Bacteroidia Bacteroidales Porphyromonadaceae Candidatus _Symbiothrix	HUT5UCF08JLH9B	10
Bacteria Bacteroidetes Bacteroidia Bacteroidales Porphyromonadaceae Candidatus _Symbiothrix	HUT5UCF08JQHKT	8
Bacteria Bacteroidetes Bacteroidia Bacteroidales Porphyromonadaceae Candidatus _Symbiothrix	HUT5UCF08JG3T2	5
Bacteria Bacteroidetes Bacteroidia Bacteroidales Porphyromonadaceae Candidatus _Symbiothrix	HUT5UCF08JKA69	9
Bacteria Bacteroidetes Bacteroidia Bacteroidales Porphyromonadaceae Candidatus _Symbiothrix	HUT5UCF08JD2OV	5
Bacteria Bacteroidetes Bacteroidia Bacteroidales Porphyromonadaceae Candidatus _Symbiothrix	HUT5UCF08JJ8ZB	26
Bacteria Bacteroidetes Bacteroidia Bacteroidales Porphyromonadaceae Candidatus _Symbiothrix	HUT5UCF08I3I4P	3
Bacteria Bacteroidetes Bacteroidia Bacteroidales Porphyromonadaceae Candidatus _Symbiothrix	HUT5UCF08I69IC	3
Bacteria Bacteroidetes Bacteroidia Bacteroidales Porphyromonadaceae Candidatus _Symbiothrix	HUT5UCF08JOWVL	2
Bacteria Bacteroidetes Bacteroidia Bacteroidales Porphyromonadaceae Candidatus _Symbiothrix	HUT5UCF08JKGXY	2
Bacteria Bacteroidetes Bacteroidia Bacteroidales Porphyromonadaceae Candidatus _Symbiothrix	HUT5UCF08JFZ2W	2
Bacteria Bacteroidetes Bacteroidia Bacteroidales Porphyromonadaceae Candidatus _Symbiothrix	HUT5UCF08I3YMA	2
Bacteria Bacteroidetes Bacteroidia Bacteroidales Porphyromonadaceae Candidatus _Symbiothrix	HUT5UCF08I91ZG	2
Bacteria Bacteroidetes Bacteroidia Bacteroidales Porphyromonadaceae Candidatus _Symbiothrix	HUT5UCF08I8DA4	2
Bacteria Bacteroidetes Bacteroidia Bacteroidales Porphyromonadaceae Candidatus _Symbiothrix	HUT5UCF08JFWW0	2
Bacteria Bacteroidetes Bacteroidia Bacteroidales Porphyromonadaceae Candidatus _Symbiothrix	HUT5UCF08JI23Z	1
Bacteria Bacteroidetes Bacteroidia Bacteroidales Porphyromonadaceae Candidatus _Symbiothrix	HUT5UCF08JJRCE	1
Bacteria Bacteroidetes Bacteroidia Bacteroidales Porphyromonadaceae Candidatus _Symbiothrix	HUT5UCF08JE7DC	1
Bacteria Bacteroidetes Bacteroidia Bacteroidales Porphyromonadaceae Candidatus _Symbiothrix	HUT5UCF08JGK9J	1
Bacteria Bacteroidetes Bacteroidia Bacteroidales Porphyromonadaceae Candidatus _Symbiothrix	HUT5UCF08JHY57	1
Bacteria Bacteroidetes Bacteroidia Bacteroidales Porphyromonadaceae Candidatus _Symbiothrix	HUT5UCF08JHJ3T	1

Bacteria Bacteroidetes Bacteroidia Bacteroidales Porphyromonadaceae Candidatus _Symbiothrix	HUT5UCF08JQT9J	1
Bacteria Bacteroidetes Bacteroidia Bacteroidales Porphyromonadaceae Candidatus _Symbiothrix	HUT5UCF08JPO47	1
Bacteria Bacteroidetes Bacteroidia Bacteroidales Porphyromonadaceae Candidatus _Symbiothrix	HUT5UCF08JOLI8	1
Bacteria Bacteroidetes Bacteroidia Bacteroidales Porphyromonadaceae Candidatus _Symbiothrix	HUT5UCF08JV24J	1
Bacteria Bacteroidetes Bacteroidia Bacteroidales Porphyromonadaceae Candidatus _Symbiothrix	HUT5UCF08JREIL	1
Bacteria Bacteroidetes Bacteroidia Bacteroidales Porphyromonadaceae Candidatus _Symbiothrix	HUT5UCF08JR8SG	1
Bacteria Bacteroidetes Bacteroidia Bacteroidales Porphyromonadaceae Candidatus _Symbiothrix	HUT5UCF08JRP1M	1
Bacteria Bacteroidetes Bacteroidia Bacteroidales Porphyromonadaceae Candidatus _Symbiothrix	HUT5UCF08JLPMQ	1
Bacteria Bacteroidetes Bacteroidia Bacteroidales Porphyromonadaceae Candidatus _Symbiothrix	HUT5UCF08JOTG0	1
Bacteria Bacteroidetes Bacteroidia Bacteroidales Porphyromonadaceae Candidatus _Symbiothrix	HUT5UCF08JMCKI	1
Bacteria Bacteroidetes Bacteroidia Bacteroidales Porphyromonadaceae Candidatus _Symbiothrix	HUT5UCF08JNITT	1
Bacteria Bacteroidetes Bacteroidia Bacteroidales Porphyromonadaceae Candidatus _Symbiothrix	HUT5UCF08JNRLR	1
Bacteria Bacteroidetes Bacteroidia Bacteroidales Porphyromonadaceae Candidatus _Symbiothrix	HUT5UCF08JOGSZ	1
Bacteria Bacteroidetes Bacteroidia Bacteroidales Porphyromonadaceae Candidatus _Symbiothrix	HUT5UCF08JAMQ8	1
Bacteria Bacteroidetes Bacteroidia Bacteroidales Porphyromonadaceae Candidatus _Symbiothrix	HUT5UCF08I9IW9	1
Bacteria Bacteroidetes Bacteroidia Bacteroidales Porphyromonadaceae Candidatus _Symbiothrix	HUT5UCF08I6HM7	1
Bacteria Bacteroidetes Bacteroidia Bacteroidales Porphyromonadaceae Candidatus _Symbiothrix	HUT5UCF08I1M3B	1
Bacteria Bacteroidetes Bacteroidia Bacteroidales Porphyromonadaceae Candidatus _Symbiothrix	HUT5UCF08I47GT	1
Bacteria Bacteroidetes Bacteroidia Bacteroidales Porphyromonadaceae Candidatus _Symbiothrix	HUT5UCF08I294R	1
Bacteria Bacteroidetes Bacteroidia Bacteroidales Porphyromonadaceae Candidatus _Symbiothrix	HUT5UCF08I25JX	1
Bacteria Bacteroidetes Bacteroidia Bacteroidales Porphyromonadaceae Candidatus _Symbiothrix	HUT5UCF08JDCZ2	1
Bacteria Bacteroidetes Bacteroidia Bacteroidales Porphyromonadaceae Candidatus _Symbiothrix	HUT5UCF08JTY8K	3
Bacteria Bacteroidetes Bacteroidia Bacteroidales Porphyromonadaceae Candidatus _Symbiothrix	HUT5UCF08I3TL6	3
Bacteria Bacteroidetes Bacteroidia Bacteroidales Porphyromonadaceae Candidatus _Symbiothrix	HUT5UCF08JDOIB	1
Bacteria Bacteroidetes Bacteroidia Bacteroidales Porphyromonadaceae Dysgonomonas	HUT5UCF08JIFN3	4
Bacteria Bacteroidetes Bacteroidia Bacteroidales Porphyromonadaceae Dysgonomonas	HUT5UCF08I236Y	2
Bacteria Bacteroidetes Bacteroidia Bacteroidales Porphyromonadaceae Dysgonomonas	HUT5UCF08JB0TM	1
Bacteria Bacteroidetes Bacteroidia Bacteroidales Porphyromonadaceae Paludibacter	HUT5UCF08JICC9	3
Bacteria Bacteroidetes Bacteroidia Bacteroidales Porphyromonadaceae Paludibacter	HUT5UCF08JJQQI	2
Bacteria Bacteroidetes Bacteroidia Bacteroidales Porphyromonadaceae Paludibacter	HUT5UCF08JFMF	5
Bacteria Bacteroidetes Bacteroidia Bacteroidales Porphyromonadaceae Paludibacter	HUT5UCF08JSB5C	4
Bacteria Bacteroidetes Bacteroidia Bacteroidales Porphyromonadaceae Parabacteroides	HUT5UCF08I0BHU	2
Bacteria Bacteroidetes Bacteroidia Bacteroidales Porphyromonadaceae Parabacteroides	HUT5UCF08JVS0Y	4

Bacteria Bacteroidetes Bacteroidia Bacteroidales Porphyromonadaceae Parabacteroides	HUT5UCF08I57K3	6
Bacteria Bacteroidetes Bacteroidia Bacteroidales Porphyromonadaceae Parabacteroides	HUT5UCF08I0BHU	10
Bacteria Bacteroidetes Bacteroidia Bacteroidales Porphyromonadaceae Proteiniphilum	HUT5UCF08JBISR	1
Bacteria Bacteroidetes Bacteroidia Bacteroidales Porphyromonadaceae Proteiniphilum	HUT5UCF08JTD88	1
Bacteria Bacteroidetes Bacteroidia Bacteroidales Porphyromonadaceae Tannerella	HUT5UCF08I7N8D	1
Bacteria Bacteroidetes Bacteroidia Bacteroidales Porphyromonadaceae Tannerella	HUT5UCF08JL8NH	3
Bacteria Bacteroidetes Bacteroidia Bacteroidales Porphyromonadaceae Tannerella	HUT5UCF08I7Q9I	9
Bacteria Bacteroidetes Bacteroidia Bacteroidales Porphyromonadaceae Tannerella	HUT5UCF08I6WT5	2
Bacteria Bacteroidetes Bacteroidia Bacteroidales Prevotellaceae Alloprevotella	HUT5UCF08I96E2	1
Bacteria Bacteroidetes Bacteroidia Bacteroidales Rikenellaceae Rs-D38_termite_group	HUT5UCF08JKXTE	1
Bacteria Bacteroidetes Bacteroidia Bacteroidales Rikenellaceae vadinBC27	HUT5UCF08JOD3R	1
Bacteria Bacteroidetes Bacteroidia Bacteroidales Rikenellaceae vadinBC27	HUT5UCF08JNMJR	1
Bacteria Bacteroidetes Bacteroidia Bacteroidales Rikenellaceae vadinBC27	HUT5UCF08JQKM7	1
Bacteria Bacteroidetes Sphingobacteriia Sphingobacteriales	HUT5UCF08I2VJ1	1
Bacteria Bacteroidetes Sphingobacteriia Sphingobacteriales Chitinophagaceae	HUT5UCF08JJ28L	2
Bacteria Bacteroidetes Sphingobacteriia Sphingobacteriales Sphingobacteriaceae Mucilaginibacter	HUT5UCF08JDBO3	1
Bacteria Candidate _division_TM7 *Candidatus* _Saccharimonas	HUT5UCF08JQCCG	2
Bacteria Candidate _division_TM7 *Candidatus* _Saccharimonas	HUT5UCF08JLL0C	6
Bacteria Candidate _division_TM7 *Candidatus* _Saccharimonas	HUT5UCF08I9115	3
Bacteria Candidate _division_TM7 *Candidatus* _Saccharimonas	HUT5UCF08JQMJ2	3
Bacteria Candidate _division_TM7 *Candidatus* _Saccharimonas	HUT5UCF08I1DTE	2
Bacteria Candidate _division_TM7 *Candidatus* _Saccharimonas	HUT5UCF08I3H06	2
Bacteria Candidate _division_TM7 *Candidatus* _Saccharimonas	HUT5UCF08JLD6M	2
Bacteria Candidate _division_TM7 *Candidatus* _Saccharimonas	HUT5UCF08JDF1D	1
Bacteria Candidate _division_TM7 *Candidatus* _Saccharimonas	HUT5UCF08JEZMC	1
Bacteria Candidate _division_TM7 *Candidatus* _Saccharimonas	HUT5UCF08JKV4T	1
Bacteria Candidate _division_TM7 *Candidatus* _Saccharimonas	HUT5UCF08JNBSL	1
Bacteria Candidate _division_TM7 *Candidatus* _Saccharimonas	HUT5UCF08JO8CC	1
Bacteria Candidate _division_TM7 *Candidatus* _Saccharimonas	HUT5UCF08JALOD	1
Bacteria Candidate _division_TM7 *Candidatus* _Saccharimonas	HUT5UCF08JBT8R	1
Bacteria Candidate _division_TM7 *Candidatus* _Saccharimonas	HUT5UCF08I3CTD	1
Bacteria Candidate _division_TM7 *Candidatus* _Saccharimonas	HUT5UCF08I3ORR	1
Bacteria Candidate _division_TM7 *Candidatus* _Saccharimonas	HUT5UCF08JDHL3	3

Bacteria Candidate _division_TM7 *Candidatus* _Saccharimonas	HUT5UCF08I6INS	2
Bacteria Candidate _division_TM7 *Candidatus* _Saccharimonas	HUT5UCF08JGRXK	1
Bacteria Candidate _division_TM7 *Candidatus* _Saccharimonas	HUT5UCF08JEOOF	1
Bacteria Candidate _division_TM7 *Candidatus* _Saccharimonas	HUT5UCF08JWBV1	1
Bacteria Candidate _division_TM7 *Candidatus* _Saccharimonas	HUT5UCF08JVDHK	1
Bacteria Candidate _division_TM7 *Candidatus* _Saccharimonas	HUT5UCF08JPZHA	1
Bacteria Candidate _division_TM7 *Candidatus* _Saccharimonas	HUT5UCF08JQ3J9	1
Bacteria Candidate _division_TM7 *Candidatus* _Saccharimonas	HUT5UCF08JRY0D	1
Bacteria Candidate _division_TM7 *Candidatus* _Saccharimonas	HUT5UCF08JS7MN	1
Bacteria Candidate _division_TM7 *Candidatus* _Saccharimonas	HUT5UCF08JEEWX	1
Bacteria Candidate _division_TM7 *Candidatus* _Saccharimonas	HUT5UCF08I24W7	1
Bacteria Candidate _division_TM7 *Candidatus* _Saccharimonas	HUT5UCF08JL1TV	2
Bacteria Candidate _division_TM7 *Candidatus* _Saccharimonas	HUT5UCF08I23KY	3
Bacteria Candidate _division_TM7 *Candidatus* _Saccharimonas	HUT5UCF08JPH2L	1
Bacteria Candidate _division_TM7 *Candidatus* _Saccharimonas	HUT5UCF08JA3BH	1
Bacteria Cyanobacteria Melainabacteria Gastranaerophilales	HUT5UCF08I98SB	1
Bacteria Cyanobacteria Melainabacteria Gastranaerophilales	HUT5UCF08JEI9X	2
Bacteria Cyanobacteria Melainabacteria Gastranaerophilales	HUT5UCF08JRJ48	1
Bacteria Cyanobacteria Melainabacteria Gastranaerophilales	HUT5UCF08I47O7	1
Bacteria Cyanobacteria Melainabacteria Gastranaerophilales	HUT5UCF08I527B	1
Bacteria Elusimicrobia Elusimicrobia Endomicrobia	HUT5UCF08JTYCN	1
Bacteria Elusimicrobia Elusimicrobia Endomicrobia	HUT5UCF08I62MC	6
Bacteria Elusimicrobia Elusimicrobia Endomicrobia	HUT5UCF08I1AKQ	4
Bacteria Elusimicrobia Elusimicrobia Endomicrobia	HUT5UCF08I2S9B	5
Bacteria Firmicutes Bacilli Bacillales Bacillaceae Bacillus	*HUT5UCF08I2KY9*	*4*
Bacteria Firmicutes Bacilli Bacillales Family_XII *Exiguobacterium*	HUT5UCF08JTFJA	1
Bacteria Firmicutes Bacilli Bacillales Staphylococcaceae Staphylococcus	HUT5UCF08JETFB	1
Bacteria Firmicutes Bacilli Lactobacillales Leuconostocaceae Leuconostoc	HUT5UCF08JHZT9	1
Bacteria Firmicutes Bacilli Lactobacillales Leuconostocaceae Leuconostoc	HUT5UCF08JEBCJ	1
Bacteria Firmicutes Bacilli Lactobacillales Streptococcaceae Lactococcus	HUT5UCF08I9A39	2
Bacteria Firmicutes Bacilli Lactobacillales Streptococcaceae Streptococcus	HUT5UCF08I99C6	3
Bacteria Firmicutes Clostridia Clostridiales Christensenellaceae	HUT5UCF08JKLYE	1
Bacteria Firmicutes Clostridia Clostridiales Christensenellaceae Christensenella	HUT5UCF08JRMPQ	1

Bacteria Firmicutes Clostridia Clostridiales Christensenellaceae Christensenella	HUT5UCF08I4230	1
Bacteria Firmicutes Clostridia Clostridiales Defluviitaleaceae Incertae_Sedis	HUT5UCF08JRG02	1
Bacteria Firmicutes Clostridia Clostridiales Defluviitaleaceae Incertae_Sedis	HUT5UCF08JB9R6	1
Bacteria Firmicutes Clostridia Clostridiales Defluviitaleaceae Incertae_Sedis	HUT5UCF08I7W33	1
Bacteria Firmicutes Clostridia Clostridiales Defluviitaleaceae Incertae_Sedis	HUT5UCF08I2SDO	1
Bacteria Firmicutes Clostridia Clostridiales Defluviitaleaceae Incertae_Sedis	HUT5UCF08I3VKK	1
Bacteria Firmicutes Clostridia Clostridiales Family_XIII uncultured	HUT5UCF08JS0LZ	3
Bacteria Firmicutes Clostridia Clostridiales Family_XIII uncultured	HUT5UCF08JIHFA	1
Bacteria Firmicutes Clostridia Clostridiales Family_XIII uncultured	HUT5UCF08I84TB	6
Bacteria Firmicutes Clostridia Clostridiales Family_XIII uncultured	HUT5UCF08JHBW1	3
Bacteria Firmicutes Clostridia Clostridiales Family_XIII uncultured	HUT5UCF08JLW6D	3
Bacteria Firmicutes Clostridia Clostridiales Family_XIII uncultured	HUT5UCF08JTBOX	3
Bacteria Firmicutes Clostridia Clostridiales Family_XIII uncultured	HUT5UCF08JPNVE	1
Bacteria Firmicutes Clostridia Clostridiales Family_XIII uncultured	HUT5UCF08JGMME	5
Bacteria Firmicutes Clostridia Clostridiales Family_XIII uncultured	HUT5UCF08JDF16	5
Bacteria Firmicutes Clostridia Clostridiales Family_XIII uncultured	HUT5UCF08JDHZT	5
Bacteria Firmicutes Clostridia Clostridiales Family_XIII uncultured	HUT5UCF08JACPX	4
Bacteria Firmicutes Clostridia Clostridiales Family_XIII uncultured	HUT5UCF08JG6TI	3
Bacteria Firmicutes Clostridia Clostridiales Family_XIII uncultured	HUT5UCF08I7YJ5	3
Bacteria Firmicutes Clostridia Clostridiales Family_XIII uncultured	HUT5UCF08JS9E0	3
Bacteria Firmicutes Clostridia Clostridiales Family_XIII uncultured	HUT5UCF08JIG26	3
Bacteria Firmicutes Clostridia Clostridiales Family_XIII uncultured	HUT5UCF08JHNQQ	3
Bacteria Firmicutes Clostridia Clostridiales Family_XIII uncultured	HUT5UCF08I3YI0	2
Bacteria Firmicutes Clostridia Clostridiales Family_XIII uncultured	HUT5UCF08JNWOE	2
Bacteria Firmicutes Clostridia Clostridiales Family_XIII uncultured	HUT5UCF08JGZR6	2
Bacteria Firmicutes Clostridia Clostridiales Family_XIII uncultured	HUT5UCF08JG28C	2
Bacteria Firmicutes Clostridia Clostridiales Family_XIII uncultured	HUT5UCF08I7R9F	2
Bacteria Firmicutes Clostridia Clostridiales Family_XIII uncultured	HUT5UCF08I9NR8	2
Bacteria Firmicutes Clostridia Clostridiales Family_XIII uncultured	HUT5UCF08I61VK	2
Bacteria Firmicutes Clostridia Clostridiales Family_XIII uncultured	HUT5UCF08JD3MX	2
Bacteria Firmicutes Clostridia Clostridiales Family_XIII uncultured	HUT5UCF08JFIN1	1
Bacteria Firmicutes Clostridia Clostridiales Family_XIII uncultured	HUT5UCF08JLV1F	1
Bacteria Firmicutes Clostridia Clostridiales Family_XIII uncultured	HUT5UCF08JJP1F	1

Bacteria	*Firmicutes*	*Clostridia*	*Clostridiales*	Family_XIII	uncultured	HUT5UCF08JIP3D	1
Bacteria	*Firmicutes*	*Clostridia*	*Clostridiales*	Family_XIII	uncultured	HUT5UCF08JI29K	1
Bacteria	*Firmicutes*	*Clostridia*	*Clostridiales*	Family_XIII	uncultured	HUT5UCF08JVDOT	1
Bacteria	*Firmicutes*	*Clostridia*	*Clostridiales*	Family_XIII	uncultured	HUT5UCF08JET5V	1
Bacteria	*Firmicutes*	*Clostridia*	*Clostridiales*	Family_XIII	uncultured	HUT5UCF08JHZL1	1
Bacteria	*Firmicutes*	*Clostridia*	*Clostridiales*	Family_XIII	uncultured	HUT5UCF08JHIH8	1
Bacteria	*Firmicutes*	*Clostridia*	*Clostridiales*	Family_XIII	uncultured	HUT5UCF08JTVTD	1
Bacteria	*Firmicutes*	*Clostridia*	*Clostridiales*	Family_XIII	uncultured	HUT5UCF08JPLAT	1
Bacteria	*Firmicutes*	*Clostridia*	*Clostridiales*	Family_XIII	uncultured	HUT5UCF08JVSB0	1
Bacteria	*Firmicutes*	*Clostridia*	*Clostridiales*	Family_XIII	uncultured	HUT5UCF08JQ8TF	1
Bacteria	*Firmicutes*	*Clostridia*	*Clostridiales*	Family_XIII	uncultured	HUT5UCF08JTBBC	1
Bacteria	*Firmicutes*	*Clostridia*	*Clostridiales*	Family_XIII	uncultured	HUT5UCF08JTLAH	1
Bacteria	*Firmicutes*	*Clostridia*	*Clostridiales*	Family_XIII	uncultured	HUT5UCF08JKRZA	1
Bacteria	*Firmicutes*	*Clostridia*	*Clostridiales*	Family_XIII	uncultured	HUT5UCF08JKIVC	1
Bacteria	*Firmicutes*	*Clostridia*	*Clostridiales*	Family_XIII	uncultured	HUT5UCF08JKTLT	1
Bacteria	*Firmicutes*	*Clostridia*	*Clostridiales*	Family_XIII	uncultured	HUT5UCF08JNM7I	1
Bacteria	*Firmicutes*	*Clostridia*	*Clostridiales*	Family_XIII	uncultured	HUT5UCF08JOEOG	1
Bacteria	*Firmicutes*	*Clostridia*	*Clostridiales*	Family_XIII	uncultured	HUT5UCF08JLWQ9	1
Bacteria	*Firmicutes*	*Clostridia*	*Clostridiales*	Family_XIII	uncultured	HUT5UCF08I5241	1
Bacteria	*Firmicutes*	*Clostridia*	*Clostridiales*	Family_XIII	uncultured	HUT5UCF08I5F2J	1
Bacteria	*Firmicutes*	*Clostridia*	*Clostridiales*	Family_XIII	uncultured	HUT5UCF08I77M2	1
Bacteria	*Firmicutes*	*Clostridia*	*Clostridiales*	Family_XIII	uncultured	HUT5UCF08I75FN	1
Bacteria	*Firmicutes*	*Clostridia*	*Clostridiales*	Family_XIII	uncultured	HUT5UCF08I9T58	1
Bacteria	*Firmicutes*	*Clostridia*	*Clostridiales*	Family_XIII	uncultured	HUT5UCF08JA8RR	1
Bacteria	*Firmicutes*	*Clostridia*	*Clostridiales*	Family_XIII	uncultured	HUT5UCF08I7J3Y	1
Bacteria	*Firmicutes*	*Clostridia*	*Clostridiales*	Family_XIII	uncultured	HUT5UCF08I2KPQ	1
Bacteria	*Firmicutes*	*Clostridia*	*Clostridiales*	Family_XIII	uncultured	HUT5UCF08JJWHW	1
Bacteria	*Firmicutes*	*Clostridia*	*Clostridiales*	Family_XIII	uncultured	HUT5UCF08JQ4CT	3
Bacteria	*Firmicutes*	*Clostridia*	*Clostridiales*	Family_XIII	uncultured	HUT5UCF08I5E1O	1
Bacteria	*Firmicutes*	*Clostridia*	*Clostridiales*	Family_XIII	uncultured	HUT5UCF08JF1YE	1
Bacteria	*Firmicutes*	*Clostridia*	*Clostridiales*	Family_XIII	uncultured	HUT5UCF08JMMBN	1
Bacteria	*Firmicutes*	*Clostridia*	*Clostridiales*	Family_XIII	uncultured	HUT5UCF08JMTO8	1
Bacteria	*Firmicutes*	*Clostridia*	*Clostridiales*	Family_XIII	uncultured	HUT5UCF08JDIID	1

Bacteria Firmicutes Clostridia Clostridiales	Family_XIII uncultured	HUT5UCF08JK79A	2
Bacteria Firmicutes Clostridia Clostridiales	Family_XIII uncultured	HUT5UCF08JGT5B	1
Bacteria Firmicutes Clostridia Clostridiales	Family_XIII uncultured	HUT5UCF08JJJV2	1
Bacteria Firmicutes Clostridia Clostridiales	Family_XIII uncultured	HUT5UCF08JETIM	1
Bacteria Firmicutes Clostridia Clostridiales	Family_XIII uncultured	HUT5UCF08I56SW	11
Bacteria Firmicutes Clostridia Clostridiales	Family_XIII uncultured	HUT5UCF08JLBZP	10
Bacteria Firmicutes Clostridia Clostridiales	Family_XIII uncultured	HUT5UCF08I837L	9
Bacteria Firmicutes Clostridia Clostridiales	Family_XIII uncultured	HUT5UCF08JF07F	8
Bacteria Firmicutes Clostridia Clostridiales	Family_XIII uncultured	HUT5UCF08JA6FY	6
Bacteria Firmicutes Clostridia Clostridiales	Family_XIII uncultured	HUT5UCF08I249H	5
Bacteria Firmicutes Clostridia Clostridiales	Family_XIII uncultured	HUT5UCF08I5PPN	5
Bacteria Firmicutes Clostridia Clostridiales	Family_XIII uncultured	HUT5UCF08I4KT3	5
Bacteria Firmicutes Clostridia Clostridiales	Family_XIII uncultured	HUT5UCF08I43N8	5
Bacteria Firmicutes Clostridia Clostridiales	Family_XIII uncultured	HUT5UCF08I6DQJ	3
Bacteria Firmicutes Clostridia Clostridiales	Family_XIII uncultured	HUT5UCF08I2TD8	4
Bacteria Firmicutes Clostridia Clostridiales	Family_XIII uncultured	HUT5UCF08JRH4O	4
Bacteria Firmicutes Clostridia Clostridiales	Family_XIII uncultured	HUT5UCF08I950D	3
Bacteria Firmicutes Clostridia Clostridiales	Family_XIII uncultured	HUT5UCF08JLT5K	3
Bacteria Firmicutes Clostridia Clostridiales	Family_XIII uncultured	HUT5UCF08I8Q5G	3
Bacteria Firmicutes Clostridia Clostridiales	Family_XIII uncultured	HUT5UCF08JQPTM	3
Bacteria Firmicutes Clostridia Clostridiales	Family_XIII uncultured	HUT5UCF08JCLJQ	2
Bacteria Firmicutes Clostridia Clostridiales	Family_XIII uncultured	HUT5UCF08I7V00	2
Bacteria Firmicutes Clostridia Clostridiales	Family_XIII uncultured	HUT5UCF08JDO8T	2
Bacteria Firmicutes Clostridia Clostridiales	Family_XIII uncultured	HUT5UCF08JLEKH	2
Bacteria Firmicutes Clostridia Clostridiales	Family_XIII uncultured	HUT5UCF08JDZBI	2
Bacteria Firmicutes Clostridia Clostridiales	Family_XIII uncultured	HUT5UCF08JC90X	2
Bacteria Firmicutes Clostridia Clostridiales	Family_XIII uncultured	HUT5UCF08JBP2G	2
Bacteria Firmicutes Clostridia Clostridiales	Family_XIII uncultured	HUT5UCF08JAC3T	2
Bacteria Firmicutes Clostridia Clostridiales	Family_XIII uncultured	HUT5UCF08I8GOR	2
Bacteria Firmicutes Clostridia Clostridiales	Family_XIII uncultured	HUT5UCF08I3CAG	2
Bacteria Firmicutes Clostridia Clostridiales	Family_XIII uncultured	HUT5UCF08I2ZQH	2
Bacteria Firmicutes Clostridia Clostridiales	Family_XIII uncultured	HUT5UCF08I25U6	2
Bacteria Firmicutes Clostridia Clostridiales	Family_XIII uncultured	HUT5UCF08I55WS	2

Bacteria Firmicutes Clostridia Clostridiales	Family_XIII uncultured	HUT5UCF08JBHHL	2
Bacteria Firmicutes Clostridia Clostridiales	Family_XIII uncultured	HUT5UCF08I3H0Q	2
Bacteria Firmicutes Clostridia Clostridiales	Family_XIII uncultured	HUT5UCF08I1J4B	2
Bacteria Firmicutes Clostridia Clostridiales	Family_XIII uncultured	HUT5UCF08I72LM	2
Bacteria Firmicutes Clostridia Clostridiales	Family_XIII uncultured	HUT5UCF08JIJM5	1
Bacteria Firmicutes Clostridia Clostridiales	Family_XIII uncultured	HUT5UCF08JVANW	1
Bacteria Firmicutes Clostridia Clostridiales	Family_XIII uncultured	HUT5UCF08JFVD7	1
Bacteria Firmicutes Clostridia Clostridiales	Family_XIII uncultured	HUT5UCF08JGT1H	1
Bacteria Firmicutes Clostridia Clostridiales	Family_XIII uncultured	HUT5UCF08JF5E6	1
Bacteria Firmicutes Clostridia Clostridiales	Family_XIII uncultured	HUT5UCF08JPOGV	1
Bacteria Firmicutes Clostridia Clostridiales	Family_XIII uncultured	HUT5UCF08JOWYJ	1
Bacteria Firmicutes Clostridia Clostridiales	Family_XIII uncultured	HUT5UCF08JROF0	1
Bacteria Firmicutes Clostridia Clostridiales	Family_XIII uncultured	HUT5UCF08JS81V	1
Bacteria Firmicutes Clostridia Clostridiales	Family_XIII uncultured	HUT5UCF08JT6TN	1
Bacteria Firmicutes Clostridia Clostridiales	Family_XIII uncultured	HUT5UCF08JPPHP	1
Bacteria Firmicutes Clostridia Clostridiales	Family_XIII uncultured	HUT5UCF08JPWST	1
Bacteria Firmicutes Clostridia Clostridiales	Family_XIII uncultured	HUT5UCF08JKW4H	1
Bacteria Firmicutes Clostridia Clostridiales	Family_XIII uncultured	HUT5UCF08JLNWT	1
Bacteria Firmicutes Clostridia Clostridiales	Family_XIII uncultured	HUT5UCF08JLQYR	1
Bacteria Firmicutes Clostridia Clostridiales	Family_XIII uncultured	HUT5UCF08JT3X4	1
Bacteria Firmicutes Clostridia Clostridiales	Family_XIII uncultured	HUT5UCF08JKPH4	1
Bacteria Firmicutes Clostridia Clostridiales	Family_XIII uncultured	HUT5UCF08JMTB2	1
Bacteria Firmicutes Clostridia Clostridiales	Family_XIII uncultured	HUT5UCF08JB2D4	1
Bacteria Firmicutes Clostridia Clostridiales	Family_XIII uncultured	HUT5UCF08I4S8R	1
Bacteria Firmicutes Clostridia Clostridiales	Family_XIII uncultured	HUT5UCF08I4TYC	1
Bacteria Firmicutes Clostridia Clostridiales	Family_XIII uncultured	HUT5UCF08JBG6Q	1
Bacteria Firmicutes Clostridia Clostridiales	Family_XIII uncultured	HUT5UCF08I7ZHV	1
Bacteria Firmicutes Clostridia Clostridiales	Family_XIII uncultured	HUT5UCF08I7QC1	1
Bacteria Firmicutes Clostridia Clostridiales	Family_XIII uncultured	HUT5UCF08I9NYX	1
Bacteria Firmicutes Clostridia Clostridiales	Family_XIII uncultured	HUT5UCF08I6AZ5	1
Bacteria Firmicutes Clostridia Clostridiales	Family_XIII uncultured	HUT5UCF08I52HJ	1
Bacteria Firmicutes Clostridia Clostridiales	Family_XIII uncultured	HUT5UCF08JCYLL	1
Bacteria Firmicutes Clostridia Clostridiales	Family_XIII uncultured	HUT5UCF08JDJG7	1

Bacteria Firmicutes Clostridia Clostridiales Family_XIII uncultured	HUT5UCF08JNK94	3
Bacteria Firmicutes Clostridia Clostridiales Family_XIII uncultured	HUT5UCF08JLZHU	1
Bacteria Firmicutes Clostridia Clostridiales Family_XIII uncultured	HUT5UCF08JEBSG	1
Bacteria Firmicutes Clostridia Clostridiales Family_XIII uncultured	HUT5UCF08JEVWL	1
Bacteria Firmicutes Clostridia Clostridiales Family_XIII uncultured	HUT5UCF08I35NE	1
Bacteria Firmicutes Clostridia Clostridiales Family_XIII uncultured	HUT5UCF08JNWR6	3
Bacteria Firmicutes Clostridia Clostridiales Family_XIII uncultured	HUT5UCF08JRRFL	4
Bacteria Firmicutes Clostridia Clostridiales Family_XIII uncultured	HUT5UCF08JKA91	1
Bacteria Firmicutes Clostridia Clostridiales Lachnospiraceae uncultured	HUT5UCF08I747M	1
Bacteria Firmicutes Clostridia Clostridiales Lachnospiraceae uncultured	HUT5UCF08JKR13	5
Bacteria Firmicutes Clostridia Clostridiales Lachnospiraceae uncultured	HUT5UCF08JHR26	1
Bacteria Firmicutes Clostridia Clostridiales Lachnospiraceae uncultured	HUT5UCF08JN4DP	1
Bacteria Firmicutes Clostridia Clostridiales Lachnospiraceae Blautia	HUT5UCF08I5RGD	1
Bacteria Firmicutes Clostridia Clostridiales Lachnospiraceae Blautia	HUT5UCF08JTTK0	1
Bacteria Firmicutes Clostridia Clostridiales Lachnospiraceae Incertae_Sedis	HUT5UCF08JAOSX	1
Bacteria Firmicutes Clostridia Clostridiales Ruminococcaceae uncultured	HUT5UCF08JIV5V	1
Bacteria Firmicutes Clostridia Clostridiales Ruminococcaceae uncultured	HUT5UCF08JIIHX	1
Bacteria Firmicutes Clostridia Clostridiales Ruminococcaceae uncultured	HUT5UCF08JEE27	1
Bacteria Firmicutes Clostridia Clostridiales Ruminococcaceae uncultured	HUT5UCF08JO8RC	1
Bacteria Firmicutes Clostridia Clostridiales Ruminococcaceae uncultured	HUT5UCF08JK3BY	1
Bacteria Firmicutes Clostridia Clostridiales Ruminococcaceae uncultured	HUT5UCF08I6MBG	1
Bacteria Firmicutes Clostridia Clostridiales Ruminococcaceae uncultured	HUT5UCF08I2MN1	1
Bacteria Firmicutes Clostridia Clostridiales Ruminococcaceae uncultured	HUT5UCF08I572S	1
Bacteria Firmicutes Clostridia Clostridiales Ruminococcaceae uncultured	HUT5UCF08JD54V	1
Bacteria Firmicutes Clostridia Clostridiales Ruminococcaceae uncultured	HUT5UCF08JCJ3Z	3
Bacteria Firmicutes Clostridia Clostridiales Ruminococcaceae uncultured	HUT5UCF08I6TGA	2
Bacteria Firmicutes Clostridia Clostridiales Ruminococcaceae uncultured	HUT5UCF08I69JD	1
Bacteria Firmicutes Clostridia Clostridiales Ruminococcaceae uncultured	HUT5UCF08JD23S	3
Bacteria Firmicutes Clostridia Clostridiales Ruminococcaceae uncultured	HUT5UCF08I65YT	2
Bacteria Firmicutes Clostridia Clostridiales Ruminococcaceae uncultured	HUT5UCF08I79AC	1
Bacteria Firmicutes Clostridia Clostridiales Ruminococcaceae uncultured	HUT5UCF08I615I	1
Bacteria Firmicutes Clostridia Clostridiales Ruminococcaceae Incertae_Sedis	HUT5UCF08I7GV6	2
Bacteria Firmicutes Clostridia Clostridiales Ruminococcaceae Incertae_Sedis	HUT5UCF08JOD7P	1

Bacteria Firmicutes Clostridia Clostridiales Ruminococcaceae Incertae_Sedis	HUT5UCF08I8REZ	1
Bacteria Firmicutes Clostridia Clostridiales Ruminococcaceae Incertae_Sedis	HUT5UCF08JAWKV	2
Bacteria Firmicutes Clostridia Clostridiales Ruminococcaceae Incertae_Sedis	HUT5UCF08JAJTH	1
Bacteria Firmicutes Clostridia Clostridiales Ruminococcaceae Incertae_Sedis	HUT5UCF08I3QPJ	1
Bacteria Firmicutes Clostridia Clostridiales Ruminococcaceae Incertae_Sedis	HUT5UCF08I96T7	1
Bacteria Firmicutes Clostridia Clostridiales Ruminococcaceae Incertae_Sedis	HUT5UCF08JA1M2	1
Bacteria Firmicutes Clostridia Clostridiales Ruminococcaceae Incertae_Sedis	HUT5UCF08I25BT	1
Bacteria Firmicutes Clostridia Clostridiales Ruminococcaceae Papillibacter	HUT5UCF08JT94O	4
Bacteria Firmicutes Clostridia Clostridiales Ruminococcaceae Papillibacter	HUT5UCF08JFTZ7	1
Bacteria Firmicutes Clostridia Clostridiales Ruminococcaceae Ruminococcus	HUT5UCF08I4CEL	4
Bacteria Firmicutes Clostridia Clostridiales Ruminococcaceae Ruminococcus	HUT5UCF08I87XK	1
Bacteria Firmicutes Clostridia Clostridiales Ruminococcaceae Ruminococcus	HUT5UCF08I8YQ3	1
Bacteria Firmicutes Clostridia Clostridiales Ruminococcaceae Ruminococcus	HUT5UCF08I98W2	2
Bacteria Firmicutes Clostridia Clostridiales Ruminococcaceae Subdoligranulum	HUT5UCF08JFKS7	2
Bacteria Firmicutes Clostridia Clostridiales Ruminococcaceae Subdoligranulum	HUT5UCF08JE3A1	1
Bacteria Firmicutes Clostridia Clostridiales vadinBB60	HUT5UCF08JR6DY	6
Bacteria Firmicutes Clostridia Clostridiales vadinBB60	HUT5UCF08JH5U3	4
Bacteria Firmicutes Clostridia Clostridiales vadinBB60	HUT5UCF08JV6CM	3
Bacteria Firmicutes Clostridia Clostridiales vadinBB60	HUT5UCF08JM4IA	3
Bacteria Firmicutes Clostridia Clostridiales vadinBB60	HUT5UCF08JQRHW	3
Bacteria Firmicutes Clostridia Clostridiales vadinBB60	HUT5UCF08I654Y	2
Bacteria Firmicutes Clostridia Clostridiales vadinBB60	HUT5UCF08JB2NT	2
Bacteria Firmicutes Clostridia Clostridiales vadinBB60	HUT5UCF08I36AQ	2
Bacteria Firmicutes Clostridia Clostridiales vadinBB60	HUT5UCF08I2GLF	2
Bacteria Firmicutes Clostridia Clostridiales vadinBB60	HUT5UCF08JS3TM	1
Bacteria Firmicutes Clostridia Clostridiales vadinBB60	HUT5UCF08JNCNZ	1
Bacteria Firmicutes Clostridia Clostridiales vadinBB60	HUT5UCF08JJQQV	1
Bacteria Firmicutes Clostridia Clostridiales vadinBB60	HUT5UCF08I9DZI	1
Bacteria Firmicutes Clostridia Clostridiales vadinBB60	HUT5UCF08JC2RF	1
Bacteria Firmicutes Clostridia Clostridiales vadinBB60	HUT5UCF08JN8N5	1
Bacteria Firmicutes Clostridia Clostridiales vadinBB60	HUT5UCF08I8KSF	2
Bacteria Firmicutes Clostridia Clostridiales vadinBB60	HUT5UCF08I5CNP	2
Bacteria Firmicutes Clostridia Clostridiales vadinBB60	HUT5UCF08JFO31	1

Bacteria Firmicutes Clostridia Clostridiales vadinBB60	HUT5UCF08JJ4MG	1
Bacteria Firmicutes Clostridia Clostridiales vadinBB60	HUT5UCF08JTM9X	1
Bacteria Firmicutes Clostridia Clostridiales vadinBB60	HUT5UCF08JQTNT	1
Bacteria Firmicutes Clostridia Clostridiales vadinBB60	HUT5UCF08JT9AF	1
Bacteria Firmicutes Clostridia Clostridiales vadinBB60	HUT5UCF08JTZ1X	1
Bacteria Firmicutes Clostridia Clostridiales vadinBB60	HUT5UCF08JM0TA	1
Bacteria Firmicutes Clostridia Clostridiales vadinBB60	HUT5UCF08I41WH	1
Bacteria Firmicutes Erysipelotrichia Erysipelotrichales Erysipelotrichaceae	HUT5UCF08JQQ7C	1
Bacteria Firmicutes Negativicutes Selenomonadales Veillonellaceae	HUT5UCF08I8FT9	3
Bacteria Firmicutes Negativicutes Selenomonadales Veillonellaceae	HUT5UCF08I6Z4X	1
Bacteria Planctomycetes vadinHA49	HUT5UCF08JD785	2
Bacteria Planctomycetes vadinHA49	HUT5UCF08JM44T	1
Bacteria Planctomycetes vadinHA49	HUT5UCF08JTT3L	1
Bacteria Proteobacteria Alphaproteobacteria Caulobacterales Caulobacteraceae Asticcacaulis	HUT5UCF08JF3H3	1
Bacteria Proteobacteria Alphaproteobacteria Rhizobiales uncultured	HUT5UCF08I8HQJ	3
Bacteria Proteobacteria Alphaproteobacteria Rhizobiales uncultured	HUT5UCF08JGDVW	1
Bacteria Proteobacteria Alphaproteobacteria Rhizobiales uncultured	HUT5UCF08JDVHN	3
Bacteria Proteobacteria Alphaproteobacteria Rhizobiales Beijerinckiaceae Methylocella	HUT5UCF08JN4NQ	1
Bacteria Proteobacteria Alphaproteobacteria Rhizobiales Bradyrhizobiaceae Bradyrhizobium	HUT5UCF08I3LQT	1
Bacteria Proteobacteria Alphaproteobacteria Rhizobiales Rhizobiaceae Rhizobium	HUT5UCF08JC38N	2
Bacteria Proteobacteria Alphaproteobacteria Rhizobiales Rhizobiales Incertae_Sedis Rhizomicrobium	HUT5UCF08JIK7T	1
Bacteria Proteobacteria Alphaproteobacteria Rhizobiales Xanthobacteraceae	HUT5UCF08JJ8YG	2
Bacteria Proteobacteria Alphaproteobacteria Rhizobiales Xanthobacteraceae Pseudolabrys	HUT5UCF08JGBIH	1
Bacteria Proteobacteria Alphaproteobacteria Rhodospirillales Acetobacteraceae uncultured	HUT5UCF08JOEOT	1
Bacteria Proteobacteria Alphaproteobacteria Rhodospirillales Acetobacteraceae uncultured	HUT5UCF08I51ZC	2
Bacteria Proteobacteria Alphaproteobacteria Rhodospirillales Acetobacteraceae uncultured	HUT5UCF08JM3LC	1
Bacteria Proteobacteria Alphaproteobacteria Rhodospirillales Acetobacteraceae uncultured	HUT5UCF08JPZST	1
Bacteria Proteobacteria Alphaproteobacteria Rhodospirillales Acetobacteraceae uncultured	HUT5UCF08JLTBP	1
Bacteria Proteobacteria Alphaproteobacteria Rhodospirillales Acetobacteraceae uncultured	HUT5UCF08I8SMH	1
Bacteria Proteobacteria Alphaproteobacteria Rhodospirillales Rhodospirillaceae Thalassospira	HUT5UCF08JFF0N	7
Bacteria Proteobacteria Alphaproteobacteria Rhodospirillales Rhodospirillaceae Thalassospira	HUT5UCF08JJPP8	5
Bacteria Proteobacteria Alphaproteobacteria Rhodospirillales Rhodospirillaceae Thalassospira	HUT5UCF08JGPNO	4
Bacteria Proteobacteria Alphaproteobacteria Rhodospirillales Rhodospirillaceae Thalassospira	HUT5UCF08JHUIZ	2

Bacteria Proteobacteria Alphaproteobacteria Rhodospirillales Rhodospirillaceae Thalassospira	HUT5UCF08JJAQ5	1
Bacteria Proteobacteria Alphaproteobacteria Rhodospirillales Rhodospirillaceae Thalassospira	HUT5UCF08JTGIX	1
Bacteria Proteobacteria Alphaproteobacteria Rhodospirillales Rhodospirillaceae Thalassospira	HUT5UCF08JVC1W	1
Bacteria Proteobacteria Alphaproteobacteria Rhodospirillales Rhodospirillaceae Thalassospira	HUT5UCF08JRYTC	1
Bacteria Proteobacteria Alphaproteobacteria Rhodospirillales Rhodospirillaceae Thalassospira	HUT5UCF08I6H5D	1
Bacteria Proteobacteria Alphaproteobacteria Rhodospirillales Rhodospirillaceae Thalassospira	HUT5UCF08I3N1L	6
Bacteria Proteobacteria Alphaproteobacteria Rhodospirillales Rhodospirillaceae Thalassospira	HUT5UCF08JFJ9Q	4
Bacteria Proteobacteria Alphaproteobacteria Rhodospirillales Rhodospirillaceae Thalassospira	HUT5UCF08JS6JQ	12
Bacteria Proteobacteria Alphaproteobacteria Sphingomonadales Sphingomonadaceae Novosphingobium	HUT5UCF08JEOWN	3
Bacteria Proteobacteria Alphaproteobacteria Sphingomonadales Sphingomonadaceae Sphingomonas	HUT5UCF08JQMJR	1
Bacteria Proteobacteria Alphaproteobacteria Sphingomonadales Sphingomonadaceae Sphingomonas	HUT5UCF08JPZ6L	1
Bacteria Proteobacteria Betaproteobacteria Burkholderiales Burkholderiaceae Burkholderia	HUT5UCF08JJTIB	2
Bacteria Proteobacteria Betaproteobacteria Burkholderiales Burkholderiaceae Burkholderia	HUT5UCF08JRSVQ	1
Bacteria Proteobacteria Betaproteobacteria Burkholderiales Burkholderiaceae Burkholderia	HUT5UCF08JB9NQ	1
Bacteria Proteobacteria Betaproteobacteria Burkholderiales Comamonadaceae Hydrogenophaga	HUT5UCF08I767O	2
Bacteria Proteobacteria Betaproteobacteria Burkholderiales Comamonadaceae Leptothrix	HUT5UCF08JAMTP	2
Bacteria Proteobacteria Betaproteobacteria Burkholderiales Comamonadaceae Simplicispira	HUT5UCF08JFVQ5	3
Bacteria Proteobacteria Betaproteobacteria Burkholderiales Comamonadaceae Simplicispira	HUT5UCF08JHHOR	1
Bacteria Proteobacteria Betaproteobacteria Burkholderiales Comamonadaceae Simplicispira	HUT5UCF08JKCTT	1
Bacteria Proteobacteria Betaproteobacteria Neisseriales Neisseriaceae Snodgrassella	HUT5UCF08JPK2Z	7
Bacteria Proteobacteria Betaproteobacteria Neisseriales Neisseriaceae Snodgrassella	HUT5UCF08JFVNJ	3
Bacteria Proteobacteria Betaproteobacteria Neisseriales Neisseriaceae Snodgrassella	HUT5UCF08I5ED2	6
Bacteria Proteobacteria Betaproteobacteria Rhodocyclales Rhodocyclaceae uncultured	HUT5UCF08I51NG	3
Bacteria Proteobacteria Betaproteobacteria Rhodocyclales Rhodocyclaceae uncultured	HUT5UCF08JKS3L	1
Bacteria Proteobacteria Betaproteobacteria Rhodocyclales Rhodocyclaceae uncultured	HUT5UCF08JHKTP	1
Bacteria Proteobacteria Betaproteobacteria Rhodocyclales Rhodocyclaceae uncultured	HUT5UCF08JFVLM	1
Bacteria Proteobacteria Betaproteobacteria Rhodocyclales Rhodocyclaceae uncultured	HUT5UCF08JGMCQ	1
Bacteria Proteobacteria Betaproteobacteria Rhodocyclales Rhodocyclaceae uncultured	HUT5UCF08JJFJN	1
Bacteria Proteobacteria Betaproteobacteria Rhodocyclales Rhodocyclaceae uncultured	HUT5UCF08JPZD1	1
Bacteria Proteobacteria Betaproteobacteria Rhodocyclales Rhodocyclaceae Azospira	HUT5UCF08JTGY3	8
Bacteria Proteobacteria Betaproteobacteria Rhodocyclales Rhodocyclaceae Candidatus_Accumulibacter	HUT5UCF08JLMTS	1
Bacteria Proteobacteria Betaproteobacteria Rhodocyclales Rhodocyclaceae Propionivibrio	HUT5UCF08I4C11	4
Bacteria Proteobacteria Betaproteobacteria Rhodocyclales Rhodocyclaceae Propionivibrio	HUT5UCF08JJ7P0	2

Bacteria Proteobacteria Betaproteobacteria Rhodocyclales Rhodocyclaceae Propionivibrio	HUT5UCF08I4QND	1
Bacteria Proteobacteria Betaproteobacteria Rhodocyclales Rhodocyclaceae Propionivibrio	HUT5UCF08JALT4	1
Bacteria Proteobacteria Betaproteobacteria Rhodocyclales Rhodocyclaceae Propionivibrio	HUT5UCF08I5RC2	1
Bacteria Proteobacteria Betaproteobacteria Rhodocyclales Rhodocyclaceae Propionivibrio	HUT5UCF08I6JX3	1
Bacteria Proteobacteria Betaproteobacteria Rhodocyclales Rhodocyclaceae Propionivibrio	HUT5UCF08JO2PT	3
Bacteria Proteobacteria Betaproteobacteria Rhodocyclales Rhodocyclaceae Propionivibrio	HUT5UCF08JSQG1	4
Bacteria Proteobacteria Betaproteobacteria Rhodocyclales Rhodocyclaceae uncultured	HUT5UCF08JBHLO	7
Bacteria Proteobacteria Betaproteobacteria Rhodocyclales Rhodocyclaceae Zoogloea	HUT5UCF08JMAP2	1
Bacteria Proteobacteria Deltaproteobacteria Desulfovibrionales Desulfovibrionaceae Desulfovibrio	HUT5UCF08JD7PD	8
Bacteria Proteobacteria Deltaproteobacteria Desulfovibrionales Desulfovibrionaceae uncultured	HUT5UCF08JSDMX	3
Bacteria Proteobacteria Deltaproteobacteria Desulfovibrionales Desulfovibrionaceae uncultured	HUT5UCF08I5XZC	2
Bacteria Proteobacteria Deltaproteobacteria Desulfovibrionales Desulfovibrionaceae uncultured	HUT5UCF08I4DXI	6
Bacteria Proteobacteria Deltaproteobacteria Desulfovibrionales Desulfovibrionaceae uncultured	HUT5UCF08I6I6Y	4
Bacteria Proteobacteria Deltaproteobacteria Desulfovibrionales Desulfovibrionaceae uncultured	HUT5UCF08I9TFK	10
Bacteria Proteobacteria Deltaproteobacteria Rs-K70_termite_group	HUT5UCF08JIAQB	3
Bacteria Proteobacteria Deltaproteobacteria Rs-K70_termite_group	HUT5UCF08I349S	1
Bacteria Proteobacteria Deltaproteobacteria Rs-K70_termite_group	HUT5UCF08I41PI	6
Bacteria Proteobacteria Deltaproteobacteria Rs-K70_termite_group	HUT5UCF08I6Q8F	6
Bacteria Proteobacteria Deltaproteobacteria Rs-K70_termite_group	HUT5UCF08I55E7	1
Bacteria Proteobacteria Deltaproteobacteria Rs-K70_termite_group	HUT5UCF08JF7H0	3
Bacteria Proteobacteria Gammaproteobacteria Chromatiales Chromatiaceae Nitrosococcus	HUT5UCF08JQ2M9	1
Bacteria Proteobacteria Gammaproteobacteria Enterobacteriales Enterobacteriaceae Cedecea	HUT5UCF08I9ZTA	1
Bacteria Proteobacteria Gammaproteobacteria Enterobacteriales Enterobacteriaceae Enterobacter	HUT5UCF08JCWHK	1
Bacteria Proteobacteria Gammaproteobacteria Enterobacteriales Enterobacteriaceae Pantoea	HUT5UCF08JJIJ8	1
Bacteria Proteobacteria Gammaproteobacteria Legionellales Coxiellaceae Aquicella	HUT5UCF08JMSTJ	1
Bacteria Proteobacteria Gammaproteobacteria Pseudomonadales Moraxellaceae Acinetobacter	HUT5UCF08I3KRC	1
Bacteria Proteobacteria Gammaproteobacteria Xanthomonadales uncultured	HUT5UCF08I5UOL	1
Bacteria Proteobacteria Gammaproteobacteria Xanthomonadales uncultured	HUT5UCF08JJM42	1
Bacteria Proteobacteria Gammaproteobacteria Xanthomonadales uncultured	HUT5UCF08JCI9P	1
Bacteria Proteobacteria Gammaproteobacteria Xanthomonadales Xanthomonadaceae Dyella	HUT5UCF08JS4SB	3
Bacteria Proteobacteria Gammaproteobacteria Xanthomonadales Xanthomonadaceae Dyella	HUT5UCF08JJNSW	1
Bacteria Proteobacteria Gammaproteobacteria Xanthomonadales Xanthomonadaceae Dyella	HUT5UCF08JGEV0	1
Bacteria Proteobacteria Gammaproteobacteria Xanthomonadales Xanthomonadaceae Rudaea	HUT5UCF08JHIQP	1

Bacteria Proteobacteria Gammaproteobacteria Xanthomonadales Xanthomonadaceae Rudaea	HUT5UCF08JBST8	2
Bacteria Proteobacteria Gammaproteobacteria Xanthomonadales Xanthomonadaceae Rudaea	HUT5UCF08JL0GJ	1
Bacteria Proteobacteria Gammaproteobacteria Xanthomonadales Xanthomonadaceae Rudaea	HUT5UCF08JMEE6	1
Bacteria Spirochaetae Spirochaetes Spirochaetales Spirochaetaceae Treponema	HUT5UCF08JTFS2	1
Bacteria Spirochaetae Spirochaetes Spirochaetales Spirochaetaceae Treponema	HUT5UCF08I7G5G	3
Bacteria Spirochaetae Spirochaetes Spirochaetales Spirochaetaceae Treponema	HUT5UCF08JIE42	4
Bacteria Spirochaetae Spirochaetes Spirochaetales Spirochaetaceae Treponema	HUT5UCF08JQZI2	3
Bacteria Spirochaetae Spirochaetes Spirochaetales Spirochaetaceae Treponema	HUT5UCF08JSL24	1
Bacteria Spirochaetae Spirochaetes Spirochaetales Spirochaetaceae Treponema	HUT5UCF08JAEI7	2
Bacteria Spirochaetae Spirochaetes Spirochaetales Spirochaetaceae Treponema	HUT5UCF08JDOC7	6
Bacteria Spirochaetae Spirochaetes Spirochaetales Spirochaetaceae Treponema	HUT5UCF08JE6GN	3
Bacteria Spirochaetae Spirochaetes Spirochaetales Spirochaetaceae Treponema	HUT5UCF08JEKSL	2
Bacteria Spirochaetae Spirochaetes Spirochaetales Spirochaetaceae Treponema	HUT5UCF08JINZ4	3
Bacteria Spirochaetae Spirochaetes Spirochaetales Spirochaetaceae Treponema	HUT5UCF08JTRX6	1
Bacteria Spirochaetae Spirochaetes Spirochaetales Spirochaetaceae Treponema	HUT5UCF08JTGS5	1
Bacteria Spirochaetae Spirochaetes Spirochaetales Spirochaetaceae Treponema	HUT5UCF08I7NJS	1
Bacteria Synergistetes Synergistia Synergistales Synergistaceae uncultured	HUT5UCF08JJY0M	1
Bacteria Synergistetes Synergistia Synergistales Synergistaceae uncultured	HUT5UCF08JIOK2	4
Bacteria Synergistetes Synergistia Synergistales Synergistaceae uncultured	HUT5UCF08JAQWZ	2
*Bacteria Synergistetes Synergistia Synergistales Synergistaceae Candidatus_* Tammella	HUT5UCF08I7ZR2	2
*Bacteria Synergistetes Synergistia Synergistales Synergistaceae Candidatus_* Tammella	HUT5UCF08I5MIQ	2
*Bacteria Synergistetes Synergistia Synergistales Synergistaceae Candidatus_* Tammella	HUT5UCF08I7TSE	2
*Bacteria Synergistetes Synergistia Synergistales Synergistaceae Candidatus_* Tammella	HUT5UCF08JOJJK	1
Bacteria Tenericutes Mollicutes Mycoplasmatales Mycoplasmataceae uncutured	HUT5UCF08JIMC2	4
Bacteria Tenericutes Mollicutes Mycoplasmatales Mycoplasmataceae uncutured	HUT5UCF08I3VBN	2
Bacteria Tenericutes Mollicutes Mycoplasmatales Mycoplasmataceae uncutured	HUT5UCF08JVYBE	1
Bacteria Tenericutes Mollicutes Mycoplasmatales Mycoplasmataceae uncutured	HUT5UCF08JLVJM	4
Bacteria Tenericutes Mollicutes Mycoplasmatales Mycoplasmataceae uncutured	HUT5UCF08JNQJ6	1
Bacteria Tenericutes Mollicutes Mycoplasmatales Mycoplasmataceae uncutured	HUT5UCF08JRY2S	6
Bacteria Tenericutes Mollicutes Mycoplasmatales Mycoplasmataceae uncutured	HUT5UCF08JMKIZ	5
Bacteria Tenericutes Mollicutes Mycoplasmatales Mycoplasmataceae uncutured	HUT5UCF08JJ112	4
Bacteria Tenericutes Mollicutes Mycoplasmatales Mycoplasmataceae uncutured	HUT5UCF08I9VKY	2
Bacteria Tenericutes Mollicutes Mycoplasmatales Mycoplasmataceae uncutured	HUT5UCF08JN45K	1

Bacteria Tenericutes Mollicutes Mycoplasmatales Mycoplasmataceae	uncutured	HUT5UCF08JMI00	1
Bacteria Tenericutes Mollicutes Mycoplasmatales Mycoplasmataceae	uncutured	HUT5UCF08I4AWF	1
Bacteria Tenericutes Mollicutes Mycoplasmatales Mycoplasmataceae	uncutured	HUT5UCF08I31ZM	1
Bacteria Tenericutes Mollicutes Mycoplasmatales Mycoplasmataceae	uncutured	HUT5UCF08I2FEM	20
Bacteria Tenericutes Mollicutes Mycoplasmatales Mycoplasmataceae	uncutured	HUT5UCF08I4BWD	9
Bacteria Tenericutes Mollicutes Mycoplasmatales Mycoplasmataceae	uncutured	HUT5UCF08JJWKQ	8
