# Supplementary material for: Gut Bacterial Community of the Xylophagous Cockroaches Cryptocercus punctulatus and Parasphaeria boleiriana
Source: PLoS One. 2016 Apr 7;11(4):e0152400. doi: 10.1371/journal.pone.0152400 (PMC4824515; doi:10.1371/journal.pone.0152400)
Supplement: S2 Table — (PDF) [file pone.0152400.s003.pdf]

S2 Table. Representative OTU at 0.05 distance for *Parasphaeria.*

**Classification**	**Representative_Seq**	**OTUs**
Bacteria Acidobacteria Acidobacteria Subgroup_2	HUT5UCF07IPVMH	1
Bacteria Acidobacteria Acidobacteria Subgroup_2	HUT5UCF07H3OE0	1
Bacteria Acidobacteria Acidobacteria Subgroup_21	HUT5UCF07ID04K	1
Bacteria Acidobacteria Acidobacteria Subgroup_3 Unknown_Family *Bryobacter*	HUT5UCF07IMJS0	1
Bacteria Acidobacteria Holophagae Subgroup_23	HUT5UCF07IOLF9	1
Bacteria Actinobacteria Acidimicrobiia Acidimicrobiales	HUT5UCF07H6OQ8	1
Bacteria Actinobacteria Acidimicrobiia Acidimicrobiales Acidimicrobiaceae uncultured	HUT5UCF07IE3G1	1
Bacteria Actinobacteria Acidimicrobiia Acidimicrobiales uncultured	HUT5UCF07IIC4B	1
Bacteria Actinobacteria Actinobacteria Actinomycetales Actinomycetaceae uncultured	HUT5UCF07H1L2K	2
Bacteria Actinobacteria Actinobacteria Actinomycetales Actinomycetaceae uncultured	HUT5UCF07ILEXB	1
Bacteria Actinobacteria Actinobacteria Corynebacteriales Corynebacteriaceae Corynebacterium	HUT5UCF07IHS8N	1
Bacteria Actinobacteria Actinobacteria Corynebacteriales Mycobacteriaceae Mycobacterium	HUT5UCF07IPT0V	1
Bacteria Actinobacteria Actinobacteria Corynebacteriales Mycobacteriaceae Mycobacterium	HUT5UCF07HWTCN	1
Bacteria Actinobacteria Actinobacteria Corynebacteriales Nocardiaceae Rhodococcus	HUT5UCF07H1328	2
Bacteria Actinobacteria Actinobacteria Frankiales Frankiaceae Jatrophihabitans	HUT5UCF07IHR6Y	1
Bacteria Actinobacteria Actinobacteria Frankiales Frankiaceae Jatrophihabitans	HUT5UCF07IIYSN	1
Bacteria Actinobacteria Actinobacteria Micrococcales Microbacteriaceae Gryllotalpicola	HUT5UCF07HWDP5	1
Bacteria Actinobacteria Actinobacteria Micrococcales Microbacteriaceae Microbacterium	HUT5UCF07IAB43	1
Bacteria Actinobacteria Actinobacteria Micrococcales Microbacteriaceae Microbacterium	HUT5UCF07IBMG7	1
Bacteria Actinobacteria Actinobacteria Micrococcales Microbacteriaceae Microbacterium	HUT5UCF07IQNID	1
Bacteria Actinobacteria Actinobacteria Micrococcales Microbacteriaceae Microbacterium	HUT5UCF07H71TQ	1
Bacteria Actinobacteria Actinobacteria Micrococcales Promicromonosporaceae Cellulosimicrobium	HUT5UCF07IQ234	6
Bacteria Actinobacteria Actinobacteria Micrococcales Promicromonosporaceae Cellulosimicrobium	HUT5UCF07H9F27	4
Bacteria Actinobacteria Actinobacteria Micrococcales Promicromonosporaceae Cellulosimicrobium	HUT5UCF07H9RDY	4
Bacteria Actinobacteria Actinobacteria Micrococcales Promicromonosporaceae Cellulosimicrobium	HUT5UCF07IHRKE	1
Bacteria Actinobacteria Actinobacteria Micrococcales Promicromonosporaceae Cellulosimicrobium	HUT5UCF07IIOLP	1
Bacteria Actinobacteria Actinobacteria Micrococcales Promicromonosporaceae Cellulosimicrobium	HUT5UCF07H2KHO	1
Bacteria Actinobacteria Actinobacteria Micrococcales Promicromonosporaceae Cellulosimicrobium	HUT5UCF07H4YVF	1
Bacteria Actinobacteria Actinobacteria Micrococcales Promicromonosporaceae Cellulosimicrobium	HUT5UCF07H0TFC	1

Bacteria Actinobacteria Actinobacteria Micrococcales Ruaniaceae Ruania	HUT5UCF07IHM2B	1
Bacteria Actinobacteria Actinobacteria Propionibacteriales Nocardioidaceae Nocardioides	HUT5UCF07IRG3E	1
Bacteria Actinobacteria Actinobacteria Propionibacteriales Nocardioidaceae Nocardioides	HUT5UCF07HXHHC	1
Bacteria Actinobacteria Actinobacteria Propionibacteriales Propionibacteriaceae Propionibacterium	HUT5UCF07H5PJI	4
Bacteria Actinobacteria Actinobacteria Streptomycetales Streptomycetaceae Streptomyces	HUT5UCF07H6EZ0	2
Bacteria Actinobacteria Actinobacteria Streptomycetales Streptomycetaceae Streptomyces	HUT5UCF07IEM21	1
Bacteria Actinobacteria Actinobacteria Streptomycetales Streptomycetaceae Streptomyces	HUT5UCF07IRKV6	1
Bacteria Actinobacteria Actinobacteria Streptomycetales Streptomycetaceae Streptomyces	HUT5UCF07HYK0C	1
Bacteria Actinobacteria Actinobacteria Streptosporangiales Thermomonosporaceae Actinoallomurus	HUT5UCF07HXHOD	1
Bacteria Actinobacteria Coriobacteriia Coriobacteriales Coriobacteriaceae uncultured	HUT5UCF07H18SS	2
Bacteria Actinobacteria Coriobacteriia Coriobacteriales Coriobacteriaceae uncultured	HUT5UCF07HYY84	1
Bacteria Actinobacteria Thermoleophilia Solirubrobacterales uncultured	HUT5UCF07H9TQ5	1
Bacteria Bacteroidete sBacteroidia Bacteroidales Porphyromonadaceae Paludibacter	HUT5UCF07H501T	3
Bacteria Bacteroidetes Bacteroidia Bacteroidales Bacteroidaceae Bacteroides	HUT5UCF07IPZA8	1
Bacteria Bacteroidetes Bacteroidia Bacteroidales Bacteroidaceae Bacteroides	HUT5UCF07ILF0D	1
Bacteria Bacteroidetes Bacteroidia Bacteroidales Bacteroidaceae Bacteroides	HUT5UCF07H0ZLM	1
Bacteria Bacteroidetes Bacteroidia Bacteroidales COB_P4-1_termite_group	HUT5UCF07H02L2	1
Bacteria Bacteroidetes Bacteroidia Bacteroidales CR-115	HUT5UCF07H10RG	2
Bacteria Bacteroidetes Bacteroidia Bacteroidales CR-115	HUT5UCF07IP3F0	1
Bacteria Bacteroidetes Bacteroidia Bacteroidales M2PB4-65_termite_group	HUT5UCF07H024G	2
Bacteria Bacteroidetes Bacteroidia Bacteroidales M2PB4-65_termite_group	HUT5UCF07HVO98	1
Bacteria Bacteroidetes Bacteroidia Bacteroidales Marinilabiaceae uncultured	HUT5UCF07H2C6Q	2
Bacteria Bacteroidetes Bacteroidia Bacteroidales Marinilabiaceae uncultured	HUT5UCF07H0IAN	1
Bacteria Bacteroidetes Bacteroidia Bacteroidales Porphyromonadaceae Butyricimonas	HUT5UCF07HU5QH	2
Bacteria Bacteroidetes Bacteroidia Bacteroidales Porphyromonadaceae Butyricimonas	HUT5UCF07IGIJS	2
Bacteria Bacteroidetes Bacteroidia Bacteroidales Porphyromonadaceae Butyricimonas	HUT5UCF07IEB7G	2
Bacteria Bacteroidetes Bacteroidia Bacteroidales Porphyromonadaceae Butyricimonas	HUT5UCF07H1DT9	1
Bacteria Bacteroidetes Bacteroidia Bacteroidales Porphyromonadaceae Candidatus _Symbiothrix	HUT5UCF07IJN7T	7
Bacteria Bacteroidetes Bacteroidia Bacteroidales Porphyromonadaceae Candidatus _Symbiothrix	HUT5UCF07IFGR8	6
Bacteria Bacteroidetes Bacteroidia Bacteroidales Porphyromonadaceae Candidatus _Symbiothrix	HUT5UCF07IG0SS	1
Bacteria Bacteroidetes Bacteroidia Bacteroidales Porphyromonadaceae Candidatus _Symbiothrix	HUT5UCF07IFFDV	1
Bacteria Bacteroidetes Bacteroidia Bacteroidales Porphyromonadaceae Candidatus _Symbiothrix	HUT5UCF07INJBJ	1
Bacteria Bacteroidetes Bacteroidia Bacteroidales Porphyromonadaceae Candidatus _Symbiothrix	HUT5UCF07H5R4T	1

Bacteria Bacteroidetes Bacteroidia Bacteroidales Porphyromonadaceae Dysgonomonas	HUT5UCF07H9TOC	2
Bacteria Bacteroidetes Bacteroidia Bacteroidales Porphyromonadaceae Dysgonomonas	HUT5UCF07IK9GI	1
Bacteria Bacteroidetes Bacteroidia Bacteroidales Porphyromonadaceae Dysgonomonas	HUT5UCF07HXCHQ	1
Bacteria Bacteroidetes Bacteroidia Bacteroidales Porphyromonadaceae Dysgonomonas	HUT5UCF07HWHC5	1
Bacteria Bacteroidetes Bacteroidia Bacteroidales Porphyromonadaceae Paludibacter	HUT5UCF07IIM8G	1
Bacteria Bacteroidetes Bacteroidia Bacteroidales Porphyromonadaceae Paludibacter	HUT5UCF07IMKXC	5
Bacteria Bacteroidetes Bacteroidia Bacteroidales Porphyromonadaceae Paludibacter	HUT5UCF07ICKAV	3
Bacteria Bacteroidetes Bacteroidia Bacteroidales Porphyromonadaceae Paludibacter	HUT5UCF07HZLTJ	3
Bacteria Bacteroidetes Bacteroidia Bacteroidales Porphyromonadaceae Paludibacter	HUT5UCF07IETB3	2
Bacteria Bacteroidetes Bacteroidia Bacteroidales Porphyromonadaceae Paludibacter	HUT5UCF07H4JBE	2
Bacteria Bacteroidetes Bacteroidia Bacteroidales Porphyromonadaceae Paludibacter	HUT5UCF07HWG0A	2
Bacteria Bacteroidetes Bacteroidia Bacteroidales Porphyromonadaceae Paludibacter	HUT5UCF07IEI2X	1
Bacteria Bacteroidetes Bacteroidia Bacteroidales Porphyromonadaceae Paludibacter	HUT5UCF07IBCH8	1
Bacteria Bacteroidetes Bacteroidia Bacteroidales Porphyromonadaceae Paludibacter	HUT5UCF07IK05E	1
Bacteria Bacteroidetes Bacteroidia Bacteroidales Porphyromonadaceae Paludibacter	HUT5UCF07H39P9	1
Bacteria Bacteroidetes Bacteroidia Bacteroidales Porphyromonadaceae Paludibacter	HUT5UCF07H9YTF	1
Bacteria Bacteroidetes Bacteroidia Bacteroidales Porphyromonadaceae Paludibacter	HUT5UCF07H9TLT	1
Bacteria Bacteroidetes Bacteroidia Bacteroidales Porphyromonadaceae Paludibacter	HUT5UCF07H42ZY	1
Bacteria Bacteroidetes Bacteroidia Bacteroidales Porphyromonadaceae Parabacteroides	HUT5UCF07H7J77	5
Bacteria Bacteroidetes Bacteroidia Bacteroidales Porphyromonadaceae Parabacteroides	HUT5UCF07H229D	3
Bacteria Bacteroidetes Bacteroidia Bacteroidales Porphyromonadaceae Parabacteroides	HUT5UCF07H4ED1	2
Bacteria Bacteroidetes Bacteroidia Bacteroidales Porphyromonadaceae Parabacteroides	HUT5UCF07HZ78I	1
Bacteria Bacteroidetes Bacteroidia Bacteroidales Porphyromonadaceae Parabacteroides	HUT5UCF07IN6TQ	1
Bacteria Bacteroidetes Bacteroidia Bacteroidales Porphyromonadaceae Parabacteroides	HUT5UCF07IPPNP	1
Bacteria Bacteroidetes Bacteroidia Bacteroidales Porphyromonadaceae Proteiniphilum	HUT5UCF07IG7JS	4
Bacteria Bacteroidetes Bacteroidia Bacteroidales Porphyromonadaceae uncultured	HUT5UCF07IEGIH	3
Bacteria Bacteroidetes Bacteroidia Bacteroidales Porphyromonadaceae uncultured	HUT5UCF07IAH1Q	1
Bacteria Bacteroidetes Bacteroidia Bacteroidales Porphyromonadaceae uncultured	HUT5UCF07ICNKX	1
Bacteria Bacteroidetes Bacteroidia Bacteroidales Porphyromonadaceae uncultured	HUT5UCF07IHXAR	1
Bacteria Bacteroidetes Bacteroidia Bacteroidales Porphyromonadaceae uncultured	HUT5UCF07IJ3T9	1
Bacteria Bacteroidetes Bacteroidia Bacteroidales Porphyromonadaceae uncultured	HUT5UCF07H3OVS	1
Bacteria Bacteroidetes Bacteroidia Bacteroidales Porphyromonadaceae uncultured	HUT5UCF07H5CXD	5
Bacteria Bacteroidetes Bacteroidia Bacteroidales Prevotellaceae Alloprevotella	HUT5UCF07H9FZP	1

Bacteria Bacteroidetes Bacteroidia Bacteroidales Rikenellaceae Alistipes	HUT5UCF07IBV72	1
Bacteria Bacteroidetes Bacteroidia Bacteroidales Rikenellaceae Alistipes	HUT5UCF07IDT3L	3
Bacteria Bacteroidetes Bacteroidia Bacteroidales Rikenellaceae Alistipes	HUT5UCF07H43DJ	2
Bacteria Bacteroidetes Bacteroidia Bacteroidales Rikenellaceae Alistipes	HUT5UCF07H44QQ	2
Bacteria Bacteroidetes Bacteroidia Bacteroidales Rikenellaceae Alistipes	HUT5UCF07H54NR	2
Bacteria Bacteroidetes Bacteroidia Bacteroidales Rikenellaceae Alistipes	HUT5UCF07H48B5	2
Bacteria Bacteroidetes Bacteroidia Bacteroidales Rikenellaceae Alistipes	HUT5UCF07IELNB	1
Bacteria Bacteroidetes Bacteroidia Bacteroidales Rikenellaceae Alistipes	HUT5UCF07IGA2J	1
Bacteria Bacteroidetes Bacteroidia Bacteroidales Rikenellaceae Alistipes	HUT5UCF07IBMLH	1
Bacteria Bacteroidetes Bacteroidia Bacteroidales Rikenellaceae Alistipes	HUT5UCF07IASVO	1
Bacteria Bacteroidetes Bacteroidia Bacteroidales Rikenellaceae Alistipes	HUT5UCF07IIET5	1
Bacteria Bacteroidetes Bacteroidia Bacteroidales Rikenellaceae Alistipes	HUT5UCF07H96OO	1
Bacteria Bacteroidetes Bacteroidia Bacteroidales Rikenellaceae Alistipes	HUT5UCF07HXJRG	1
Bacteria Bacteroidetes Bacteroidia Bacteroidales Rikenellaceae Alistipes	HUT5UCF07HZI8C	1
Bacteria Bacteroidetes Bacteroidia Bacteroidales Rikenellaceae Alistipes	HUT5UCF07HZ4KV	1
Bacteria Bacteroidetes Bacteroidia Bacteroidales Rikenellaceae Alistipes	HUT5UCF07H9YGV	1
Bacteria Bacteroidetes Bacteroidia Bacteroidales Rikenellaceae Alistipes	HUT5UCF07H20ZO	1
Bacteria Bacteroidetes Bacteroidia Bacteroidales Rikenellaceae M2PB4-61_termite_group	HUT5UCF07H3DLZ	1
Bacteria Bacteroidetes Bacteroidia Bacteroidales Rikenellaceae RC9_gut_group	HUT5UCF07IJLX6	1
Bacteria Bacteroidetes Bacteroidia Bacteroidales Rikenellaceae Rikenella	HUT5UCF07II5HK	1
Bacteria Bacteroidetes Bacteroidia Bacteroidales Rikenellaceae Rikenella	HUT5UCF07IGC6Q	1
Bacteria Bacteroidetes Bacteroidia Bacteroidales Rikenellaceae uncultured	HUT5UCF07H2YSJ	1
Bacteria Bacteroidetes Bacteroidia Bacteroidales Rikenellaceae uncultured	HUT5UCF07HX6PP	1
Bacteria Bacteroidetes Bacteroidia Bacteroidales Rikenellaceae vadinBC27	HUT5UCF07H0QVX	4
Bacteria Bacteroidetes Bacteroidia Bacteroidales Rikenellaceae vadinBC27	HUT5UCF07H8T57	3
Bacteria Bacteroidetes Bacteroidia Bacteroidales Rikenellaceae vadinBC27	HUT5UCF07IFF59	1
Bacteria Bacteroidetes Bacteroidia Bacteroidales Rs-E47_termite_group	HUT5UCF07IIXNG	3
Bacteria Bacteroidetes Bacteroidia Bacteroidales uncultured	HUT5UCF07H1M3J	2
Bacteria Bacteroidetes Bacteroidia Bacteroidales uncultured	HUT5UCF07II9R9	1
Bacteria Bacteroidetes Bacteroidia Bacteroidales vadinHA21	HUT5UCF07IK3TS	4
Bacteria Bacteroidetes Bacteroidia Bacteroidales vadinHA21	HUT5UCF07IEY32	3
Bacteria Bacteroidetes Bacteroidia Bacteroidales vadinHA21	HUT5UCF07IBAES	1
Bacteria Bacteroidetes Bacteroidia Bacteroidales vadinHA21	HUT5UCF07H8CI5	1

Bacteria Bacteroidetes Cytophagia Cytophagales Cyclobacteriaceae Nitritalea	HUT5UCF07ILE8H	1
Bacteria Bacteroidetes Cytophagia Cytophagales Cytophagaceae Dyadobacter	HUT5UCF07IIT5M	1
Bacteria Bacteroidetes Cytophagia Cytophagales Flammeovirgaceae Fulvivirga	HUT5UCF07H3KQ1	1
Bacteria Bacteroidetes Cytophagia Cytophagales Flammeovirgaceae Reichenbachiella	HUT5UCF07HW87Y	1
Bacteria Bacteroidetes Cytophagia Cytophagales Flammeovirgaceae Roseivirga	HUT5UCF07H47LI	2
Bacteria Bacteroidetes Cytophagia Order_II Rhodothermaceae uncultured	HUT5UCF07IGPNN	1
Bacteria Bacteroidetes Cytophagia Order_III uncultured	HUT5UCF07H93CF	1
Bacteria Bacteroidetes Cytophagia Order_III uncultured	HUT5UCF07H6NBP	1
Bacteria Bacteroidetes Cytophagia Order_III Unknown_Family *Gracilimonas*	HUT5UCF07H16MT	1
Bacteria Bacteroidetes Flavobacteriia Flavobacteriales Cryomorphaceae Cryomorpha	HUT5UCF07H6TNY	1
Bacteria Bacteroidetes Flavobacteriia Flavobacteriales Cryomorphaceae Owenweeksia	HUT5UCF07H4OMX	1
Bacteria Bacteroidetes Flavobacteriia Flavobacteriales Flavobacteriaceae Robiginitalea	HUT5UCF07H0CO2	2
Bacteria Bacteroidetes Flavobacteriia Flavobacteriales Flavobacteriaceae Salinimicrobium	HUT5UCF07H84H1	1
Bacteria Bacteroidetes Flavobacteriia Flavobacteriales Flavobacteriaceae Winogradskyella	HUT5UCF07H1HP1	1
Bacteria Bacteroidetes Sphingobacteriia Sphingobacteriales Chitinophagaceae uncultured	HUT5UCF07H7TBK	2
Bacteria Bacteroidetes Sphingobacteriia Sphingobacteriales Saprospiraceae uncultured	HUT5UCF07IHMTC	1
Bacteria Bacteroidetes Sphingobacteriia Sphingobacteriales Saprospiraceae uncultured	HUT5UCF07H2DTT	1
Bacteria Bacteroidetes Sphingobacteriia Sphingobacteriales Saprospiraceae uncultured	HUT5UCF07H5KSG	1
Bacteria Bacteroidetes Sphingobacteriia Sphingobacteriales Sphingobacteriaceae Sphingobacterium	HUT5UCF07IESAQ	1
Bacteria Bacteroidetes Sphingobacteriia Sphingobacteriales Sphingobacteriaceae Sphingobacterium	HUT5UCF07H3H7L	2
Bacteria Bacteroidetes Sphingobacteriia Sphingobacteriales ST-12K33	HUT5UCF07IJYIX	1
Bacteria Bacteroidetes Sphingobacteriia Sphingobacteriales WCHB1-69	HUT5UCF07H04QI	1
Bacteria Candidate _division_TM7	HUT5UCF07IASX1	1
Bacteria Candidate _division_TM7	HUT5UCF07H3DM6	1
Bacteria Candidate _division_TM7	HUT5UCF07H1CLQ	1
Bacteria Chloroflexi Anaerolineae Anaerolineales Anaerolineaceae uncultured	HUT5UCF07IGWES	1
Bacteria Chloroflexi Ardenticatenia uncultured	HUT5UCF07IK51T	1
Bacteria Deferribacteres Deferribacteres Deferribacterales Deferribacterales_Incertae_Sedis Caldithrix	HUT5UCF07H1RZH	2
Bacteria Deferribacteres Deferribacteres Deferribacterales Deferribacterales_Incertae_Sedis Caldithrix	HUT5UCF07IK2M6	1
Bacteria Deferribacteres Deferribacteres Deferribacterales Deferribacterales_Incertae_Sedis Caldithrix	HUT5UCF07ILDCG	1
Bacteria Fibrobacteres Fibrobacteria Fibrobacterales Fibrobacteraceae possible_genus_06	HUT5UCF07H0YAK	2
Bacteria Fibrobacteres Fibrobacteria Fibrobacterales uncultured	HUT5UCF07HYZGE	1
Bacteria Firmicutes Clostridia Clostridiales Lachnospiraceae Roseburia	HUT5UCF07HWEYB	1

Bacteria Firmicutes Bacilli Bacillales Bacillaceae Bacillus	HUT5UCF07H9SAP	1
Bacteria Firmicutes Bacilli Bacillales Paenibacillaceae Cohnella	HUT5UCF07IDRTE	1
Bacteria Firmicutes Bacilli Bacillales Paenibacillaceae Cohnella	HUT5UCF07IOMDD	1
Bacteria Firmicutes Bacilli Bacillales Paenibacillaceae Paenibacillus	HUT5UCF07H2ATD	1
Bacteria Firmicutes Bacilli Bacillales Paenibacillaceae Paenibacillus	HUT5UCF07H8F8A	1
Bacteria Firmicutes Bacilli Bacillales Staphylococcaceae Staphylococcus	HUT5UCF07H5V9I	1
Bacteria Firmicutes Bacilli Bacillales Staphylococcaceae Staphylococcus	HUT5UCF07HVLTZ	1
Bacteria Firmicutes Bacilli Bacillales Thermoactinomycetaceae Planifilum	HUT5UCF07IPGE8	1
Bacteria Firmicutes Bacilli Lactobacillales Enterococcaceae Enterococcus	HUT5UCF07ICR5U	3
Bacteria Firmicutes Bacilli Lactobacillales Lactobacillaceae Lactobacillus	HUT5UCF07IMZG9	1
Bacteria Firmicutes Bacilli Lactobacillales Streptococcaceae Lactococcus	HUT5UCF07IGJID	12
Bacteria Firmicutes Bacilli Lactobacillales Streptococcaceae Lactococcus	HUT5UCF07IGACN	2
Bacteria Firmicutes Bacilli Lactobacillales Streptococcaceae Lactococcus	HUT5UCF07IF9JV	1
Bacteria Firmicutes Bacilli Lactobacillales Streptococcaceae Lactococcus	HUT5UCF07H1CGG	1
Bacteria Firmicutes Clostridia Clostridiale sChristensenellaceae uncultured	HUT5UCF07H0NIK	4
Bacteria Firmicutes Clostridia Clostridiale sChristensenellaceae uncultured	HUT5UCF07IPJG1	1
Bacteria Firmicutes Clostridia Clostridiales Christensenellaceae	HUT5UCF07IPJST	1
Bacteria Firmicutes Clostridia Clostridiales Christensenellaceae Christensenella	HUT5UCF07H156K	1
Bacteria Firmicutes Clostridia Clostridiales Christensenellaceae Christensenella	HUT5UCF07INWNV	1
Bacteria Firmicutes Clostridia Clostridiales Christensenellaceae Christensenella	HUT5UCF07IH3LE	1
Bacteria Firmicutes Clostridia Clostridiales Christensenellaceae Christensenella	HUT5UCF07IHD6H	1
Bacteria Firmicutes Clostridia Clostridiales Christensenellaceae Christensenella	HUT5UCF07H34KM	1
Bacteria Firmicutes Clostridia Clostridiales Christensenellaceae Christensenella	HUT5UCF07H5G7T	1
Bacteria Firmicutes Clostridia Clostridiales Christensenellaceae Christensenella	HUT5UCF07H18KP	1
Bacteria Firmicutes Clostridia Clostridiales Christensenellaceae uncultured	HUT5UCF07IHFBA	2
Bacteria Firmicutes Clostridia Clostridiales Christensenellaceae uncultured	HUT5UCF07H8ZIG	5
Bacteria Firmicutes Clostridia Clostridiales Christensenellaceae uncultured	HUT5UCF07H63LA	4
Bacteria Firmicutes Clostridia Clostridiales Christensenellaceae uncultured	HUT5UCF07H818A	4
Bacteria Firmicutes Clostridia Clostridiales Christensenellaceae uncultured	HUT5UCF07IO6T3	4
Bacteria Firmicutes Clostridia Clostridiales Christensenellaceae uncultured	HUT5UCF07H34L2	3
Bacteria Firmicutes Clostridia Clostridiales Christensenellaceae uncultured	HUT5UCF07IPHDW	3
Bacteria Firmicutes Clostridia Clostridiales Christensenellaceae uncultured	HUT5UCF07H6T9S	3
Bacteria Firmicutes Clostridia Clostridiales Christensenellaceae uncultured	HUT5UCF07IFDT4	3

Bacteria Firmicutes Clostridia Clostridiales Christensenellaceae	uncultured	HUT5UCF07HZD3D	3
Bacteria Firmicutes Clostridia Clostridiales Christensenellaceae	uncultured	HUT5UCF07H919K	2
Bacteria Firmicutes Clostridia Clostridiales Christensenellaceae	uncultured	HUT5UCF07ID95C	2
Bacteria Firmicutes Clostridia Clostridiales Christensenellaceae	uncultured	HUT5UCF07H4CP8	2
Bacteria Firmicutes Clostridia Clostridiales Christensenellaceae	uncultured	HUT5UCF07H2KQF	2
Bacteria Firmicutes Clostridia Clostridiales Christensenellaceae	uncultured	HUT5UCF07H1JZE	2
Bacteria Firmicutes Clostridia Clostridiales Christensenellaceae	uncultured	HUT5UCF07HXWG2	2
Bacteria Firmicutes Clostridia Clostridiales Christensenellaceae	uncultured	HUT5UCF07H7XWM	2
Bacteria Firmicutes Clostridia Clostridiales Christensenellaceae	uncultured	HUT5UCF07H0W9H	2
Bacteria Firmicutes Clostridia Clostridiales Christensenellaceae	uncultured	HUT5UCF07IIRWK	2
Bacteria Firmicutes Clostridia Clostridiales Christensenellaceae	uncultured	HUT5UCF07H0ERK	2
Bacteria Firmicutes Clostridia Clostridiales Christensenellaceae	uncultured	HUT5UCF07HW7P8	2
Bacteria Firmicutes Clostridia Clostridiales Christensenellaceae	uncultured	HUT5UCF07H45NV	2
Bacteria Firmicutes Clostridia Clostridiales Christensenellaceae	uncultured	HUT5UCF07H8NVF	2
Bacteria Firmicutes Clostridia Clostridiales Christensenellaceae	uncultured	HUT5UCF07H2C01	2
Bacteria Firmicutes Clostridia Clostridiales Christensenellaceae	uncultured	HUT5UCF07IFJKG	1
Bacteria Firmicutes Clostridia Clostridiales Christensenellaceae	uncultured	HUT5UCF07IF4KT	1
Bacteria Firmicutes Clostridia Clostridiales Christensenellaceae	uncultured	HUT5UCF07IFWHR	1
Bacteria Firmicutes Clostridia Clostridiales Christensenellaceae	uncultured	HUT5UCF07IG2IZ	1
Bacteria Firmicutes Clostridia Clostridiales Christensenellaceae	uncultured	HUT5UCF07IENOL	1
Bacteria Firmicutes Clostridia Clostridiales Christensenellaceae	uncultured	HUT5UCF07IAD0G	1
Bacteria Firmicutes Clostridia Clostridiales Christensenellaceae	uncultured	HUT5UCF07HZN1T	1
Bacteria Firmicutes Clostridia Clostridiales Christensenellaceae	uncultured	HUT5UCF07IF303	1
Bacteria Firmicutes Clostridia Clostridiales Christensenellaceae	uncultured	HUT5UCF07ILT1J	1
Bacteria Firmicutes Clostridia Clostridiales Christensenellaceae	uncultured	HUT5UCF07IMITB	1
Bacteria Firmicutes Clostridia Clostridiales Christensenellaceae	uncultured	HUT5UCF07INB4M	1
Bacteria Firmicutes Clostridia Clostridiales Christensenellaceae	uncultured	HUT5UCF07IRRLT	1
Bacteria Firmicutes Clostridia Clostridiales Christensenellaceae	uncultured	HUT5UCF07IPP5F	1
Bacteria Firmicutes Clostridia Clostridiales Christensenellaceae	uncultured	HUT5UCF07IIF1O	1
Bacteria Firmicutes Clostridia Clostridiales Christensenellaceae	uncultured	HUT5UCF07IHLJS	1
Bacteria Firmicutes Clostridia Clostridiales Christensenellaceae	uncultured	HUT5UCF07IJ216	1
Bacteria Firmicutes Clostridia Clostridiales Christensenellaceae	uncultured	HUT5UCF07H570A	1
Bacteria Firmicutes Clostridia Clostridiales Christensenellaceae	uncultured	HUT5UCF07H16JM	1

Bacteria Firmicutes Clostridia Clostridiales Christensenellaceae uncultured	HUT5UCF07HX3NY	1
Bacteria Firmicutes Clostridia Clostridiales Christensenellaceae uncultured	HUT5UCF07HZGXF	1
Bacteria Firmicutes Clostridia Clostridiales Christensenellaceae uncultured	HUT5UCF07H29OY	3
Bacteria Firmicutes Clostridia Clostridiales Christensenellaceae uncultured	HUT5UCF07IHY9T	3
Bacteria Firmicutes Clostridia Clostridiales Christensenellaceae uncultured	HUT5UCF07IPT9O	3
Bacteria Firmicutes Clostridia Clostridiales Christensenellaceae uncultured	HUT5UCF07IHY4V	2
Bacteria Firmicutes Clostridia Clostridiales Christensenellaceae uncultured	HUT5UCF07IH8JM	2
Bacteria Firmicutes Clostridia Clostridiales Clostridiacea e_1 *Clostridium_sensu_stricto_* 1	HUT5UCF07HYJVA	8
Bacteria Firmicutes Clostridia Clostridiales Clostridiacea e_1 *Clostridium_sensu_stricto_* 1	HUT5UCF07H6468	2
Bacteria Firmicutes Clostridia Clostridiales Clostridiacea e_1 *Clostridium_sensu_stricto_* 1	HUT5UCF07IHQP8	1
Bacteria Firmicutes Clostridia Clostridiales Clostridiacea e_1 *Clostridium_sensu_stricto_* 1	HUT5UCF07H2ENP	1
*Bacteria Firmicutes Clostridia Clostridiales Clostridiaceae_* 1 *Clostridium_sensu_stricto* _11	HUT5UCF07H5GBX	1
Bacteria Firmicutes Clostridia Clostridiales Defluviitaleaceae Incertae_Sedis	HUT5UCF07H5CFT	3
Bacteria Firmicutes Clostridia Clostridiales Defluviitaleaceae Incertae_Sedis	HUT5UCF07IFNF7	1
Bacteria Firmicutes Clostridia Clostridiales Defluviitaleaceae uncultured	HUT5UCF07IH25F	1
Bacteria Firmicutes Clostridia Clostridiales Defluviitaleaceae uncultured	HUT5UCF07HWOGR	1
Bacteria Firmicutes Clostridia Clostridiales Defluviitaleaceae uncultured	HUT5UCF07H3EB9	2
Bacteria Firmicutes Clostridia Clostridiales Eubacteriaceae Anaerofustis	HUT5UCF07H96HJ	1
Bacteria Firmicutes Clostridia Clostridiales Family_XIII uncultured	HUT5UCF07IA3BG	2
Bacteria Firmicutes Clostridia Clostridiales Family_XIII uncultured	HUT5UCF07H6D82	3
Bacteria Firmicutes Clostridia Clostridiales Family_XIII uncultured	HUT5UCF07ION9J	4
Bacteria Firmicutes Clostridia Clostridiales Family_XIII uncultured	HUT5UCF07IO09B	1
Bacteria Firmicutes Clostridia Clostridiales Family_XIII uncultured	HUT5UCF07IHCWT	1
Bacteria Firmicutes Clostridia Clostridiales Family_XIII uncultured	HUT5UCF07IL27G	1
Bacteria Firmicutes Clostridia Clostridiales Family_XIII uncultured	HUT5UCF07H8K8E	1
Bacteria Firmicutes Clostridia Clostridiales Family_XIII uncultured	HUT5UCF07H97Q4	1
Bacteria Firmicutes Clostridia Clostridiales Family_XIII uncultured	HUT5UCF07H2BXL	1
Bacteria Firmicutes Clostridia Clostridiales Family_XIII uncultured	HUT5UCF07HXGHU	1
Bacteria Firmicutes Clostridia Clostridiales Lachnospiraceae Blautia	HUT5UCF07HXH06	1
Bacteria Firmicutes Clostridia Clostridiales Lachnospiraceae Incertae_Sedis	HUT5UCF07IEIDT	2
Bacteria Firmicutes Clostridia Clostridiales Peptococcaceae uncultured	HUT5UCF07HVY2A	2
Bacteria Firmicutes Clostridia Clostridiales Peptococcaceae uncultured	HUT5UCF07IFQ9K	1
Bacteria Firmicutes Clostridia Clostridiales Peptococcaceae uncultured	HUT5UCF07IO1L0	1

Bacteria Firmicutes Clostridia Clostridiales Peptococcaceae uncultured	HUT5UCF07IROPI	1
Bacteria Firmicutes Clostridia Clostridiales Peptococcaceae uncultured	HUT5UCF07IQVN3	1
Bacteria Firmicutes Clostridia Clostridiales Peptococcaceae uncultured	HUT5UCF07H0GR6	1
Bacteria Firmicutes Clostridia Clostridiales Peptococcaceae uncultured	HUT5UCF07H54MY	3
Bacteria Firmicutes Clostridia Clostridiales Peptococcaceae uncultured	HUT5UCF07H807T	1
Bacteria Firmicutes Clostridia Clostridiales Ruminococcaceae Anaerofilum	HUT5UCF07H6XEE	1
Bacteria Firmicutes Clostridia Clostridiales Ruminococcaceae Anaerotruncus	HUT5UCF07IA1XE	1
Bacteria Firmicutes Clostridia Clostridiales Ruminococcaceae Anaerotruncus	HUT5UCF07IA3I6	1
Bacteria Firmicutes Clostridia Clostridiales Ruminococcaceae Anaerotruncus	HUT5UCF07IGW8N	1
Bacteria Firmicutes Clostridia Clostridiales Ruminococcaceae Anaerotruncus	HUT5UCF07H0HCD	1
Bacteria Firmicutes Clostridia Clostridiales Ruminococcaceae Hydrogenoanaerobacterium	HUT5UCF07ILEEW	1
Bacteria Firmicutes Clostridia Clostridiales Ruminococcaceae Papillibacter	HUT5UCF07IHWK7	3
Bacteria Firmicutes Clostridia Clostridiales Ruminococcaceae Ruminococcus	HUT5UCF07HZHWY	1
Bacteria Firmicutes Clostridia Clostridiales Ruminococcaceae Ruminococcus	HUT5UCF07HVE34	1
Bacteria Firmicutes Clostridia Clostridiales Ruminococcaceae Saccharofermentans	HUT5UCF07IDMT1	1
Bacteria Firmicutes Clostridia Clostridiales Ruminococcaceae uncultured	HUT5UCF07H26Y2	4
Bacteria Firmicutes Clostridia Clostridiales Ruminococcaceae uncultured	HUT5UCF07H71T7	4
Bacteria Firmicutes Clostridia Clostridiales Ruminococcaceae uncultured	HUT5UCF07H882Y	3
Bacteria Firmicutes Clostridia Clostridiales Ruminococcaceae uncultured	HUT5UCF07IBQYY	3
Bacteria Firmicutes Clostridia Clostridiales Ruminococcaceae uncultured	HUT5UCF07ICM8L	3
Bacteria Firmicutes Clostridia Clostridiales Ruminococcaceae uncultured	HUT5UCF07H7MQI	2
Bacteria Firmicutes Clostridia Clostridiales Ruminococcaceae uncultured	HUT5UCF07H4QA4	2
Bacteria Firmicutes Clostridia Clostridiales Ruminococcaceae uncultured	HUT5UCF07HVSOA	2
Bacteria Firmicutes Clostridia Clostridiales Ruminococcaceae uncultured	HUT5UCF07H30EH	2
Bacteria Firmicutes Clostridia Clostridiales Ruminococcaceae uncultured	HUT5UCF07H7OMY	2
Bacteria Firmicutes Clostridia Clostridiales Ruminococcaceae uncultured	HUT5UCF07HX78L	2
Bacteria Firmicutes Clostridia Clostridiales Ruminococcaceae uncultured	HUT5UCF07IDAQA	2
Bacteria Firmicutes Clostridia Clostridiales Ruminococcaceae uncultured	HUT5UCF07H2N66	2
Bacteria Firmicutes Clostridia Clostridiales Ruminococcaceae uncultured	HUT5UCF07H2NG8	2
Bacteria Firmicutes Clostridia Clostridiales Ruminococcaceae uncultured	HUT5UCF07H2CZ4	2
Bacteria Firmicutes Clostridia Clostridiales Ruminococcaceae uncultured	HUT5UCF07H8S6T	2
Bacteria Firmicutes Clostridia Clostridiales Ruminococcaceae uncultured	HUT5UCF07H79VV	2
Bacteria Firmicutes Clostridia Clostridiales Ruminococcaceae uncultured	HUT5UCF07ICLTT	2

Bacteria Firmicutes Clostridia Clostridiales Ruminococcaceae	uncultured	HUT5UCF07IFPSX	1
Bacteria Firmicutes Clostridia Clostridiales Ruminococcaceae	uncultured	HUT5UCF07IFZYI	1
Bacteria Firmicutes Clostridia Clostridiales Ruminococcaceae	uncultured	HUT5UCF07IEJYB	1
Bacteria Firmicutes Clostridia Clostridiales Ruminococcaceae	uncultured	HUT5UCF07IG7KW	1
Bacteria Firmicutes Clostridia Clostridiales Ruminococcaceae	uncultured	HUT5UCF07IFR3R	1
Bacteria Firmicutes Clostridia Clostridiales Ruminococcaceae	uncultured	HUT5UCF07IFO3A	1
Bacteria Firmicutes Clostridia Clostridiales Ruminococcaceae	uncultured	HUT5UCF07IASYO	1
Bacteria Firmicutes Clostridia Clostridiales Ruminococcaceae	uncultured	HUT5UCF07HZK4I	1
Bacteria Firmicutes Clostridia Clostridiales Ruminococcaceae	uncultured	HUT5UCF07HZSF5	1
Bacteria Firmicutes Clostridia Clostridiales Ruminococcaceae	uncultured	HUT5UCF07HZQYK	1
Bacteria Firmicutes Clostridia Clostridiales Ruminococcaceae	uncultured	HUT5UCF07IA7AF	1
Bacteria Firmicutes Clostridia Clostridiales Ruminococcaceae	uncultured	HUT5UCF07ICFF9	1
Bacteria Firmicutes Clostridia Clostridiales Ruminococcaceae	uncultured	HUT5UCF07IBYYV	1
Bacteria Firmicutes Clostridia Clostridiales Ruminococcaceae	uncultured	HUT5UCF07IBOR7	1
Bacteria Firmicutes Clostridia Clostridiales Ruminococcaceae	uncultured	HUT5UCF07IOBDQ	1
Bacteria Firmicutes Clostridia Clostridiales Ruminococcaceae	uncultured	HUT5UCF07IOH24	1
Bacteria Firmicutes Clostridia Clostridiales Ruminococcaceae	uncultured	HUT5UCF07IMGH5	1
Bacteria Firmicutes Clostridia Clostridiales Ruminococcaceae	uncultured	HUT5UCF07IOXEJ	1
Bacteria Firmicutes Clostridia Clostridiales Ruminococcaceae	uncultured	HUT5UCF07IP2RL	1
Bacteria Firmicutes Clostridia Clostridiales Ruminococcaceae	uncultured	HUT5UCF07INZ26	1
Bacteria Firmicutes Clostridia Clostridiales Ruminococcaceae	uncultured	HUT5UCF07IPP4Z	1
Bacteria Firmicutes Clostridia Clostridiales Ruminococcaceae	uncultured	HUT5UCF07IQB90	1
Bacteria Firmicutes Clostridia Clostridiales Ruminococcaceae	uncultured	HUT5UCF07IRRFS	1
Bacteria Firmicutes Clostridia Clostridiales Ruminococcaceae	uncultured	HUT5UCF07IQOA3	1
Bacteria Firmicutes Clostridia Clostridiales Ruminococcaceae	uncultured	HUT5UCF07IQOT9	1
Bacteria Firmicutes Clostridia Clostridiales Ruminococcaceae	uncultured	HUT5UCF07IQ4R8	1
Bacteria Firmicutes Clostridia Clostridiales Ruminococcaceae	uncultured	HUT5UCF07IQOJK	1
Bacteria Firmicutes Clostridia Clostridiales Ruminococcaceae	uncultured	HUT5UCF07IHAI7	1
Bacteria Firmicutes Clostridia Clostridiales Ruminococcaceae	uncultured	HUT5UCF07IK4QW	1
Bacteria Firmicutes Clostridia Clostridiales Ruminococcaceae	uncultured	HUT5UCF07IKNKL	1
Bacteria Firmicutes Clostridia Clostridiales Ruminococcaceae	uncultured	HUT5UCF07IJB3C	1
Bacteria Firmicutes Clostridia Clostridiales Ruminococcaceae	uncultured	HUT5UCF07IL3Y7	1
Bacteria Firmicutes Clostridia Clostridiales Ruminococcaceae	uncultured	HUT5UCF07IL4KR	1

Bacteria Firmicutes Clostridia Clostridiales Ruminococcaceae uncultured	HUT5UCF07H8RL1	1
Bacteria Firmicutes Clostridia Clostridiales Ruminococcaceae uncultured	HUT5UCF07H930K	1
Bacteria Firmicutes Clostridia Clostridiales Ruminococcaceae uncultured	HUT5UCF07H9I8X	1
Bacteria Firmicutes Clostridia Clostridiales Ruminococcaceae uncultured	HUT5UCF07H3QML	1
Bacteria Firmicutes Clostridia Clostridiales Ruminococcaceae uncultured	HUT5UCF07H9MVH	1
Bacteria Firmicutes Clostridia Clostridiales Ruminococcaceae uncultured	HUT5UCF07H9P1V	1
Bacteria Firmicutes Clostridia Clostridiales Ruminococcaceae uncultured	HUT5UCF07H7T52	1
Bacteria Firmicutes Clostridia Clostridiales Ruminococcaceae uncultured	HUT5UCF07H1WOH	1
Bacteria Firmicutes Clostridia Clostridiales Ruminococcaceae uncultured	HUT5UCF07HWD9C	1
Bacteria Firmicutes Clostridia Clostridiales Ruminococcaceae uncultured	HUT5UCF07HU2CZ	1
Bacteria Firmicutes Clostridia Clostridiales Ruminococcaceae uncultured	HUT5UCF07H2ESI	1
Bacteria Firmicutes Clostridia Clostridiales Ruminococcaceae uncultured	HUT5UCF07HXG8R	3
Bacteria Firmicutes Clostridia Clostridiales Ruminococcaceae uncultured	HUT5UCF07H017E	3
Bacteria Firmicutes Clostridia Clostridiales Ruminococcaceae uncultured	HUT5UCF07HXAQ6	3
Bacteria Firmicutes Clostridia Clostridiales Ruminococcaceae uncultured	HUT5UCF07HUY16	3
Bacteria Firmicutes Clostridia Clostridiales Ruminococcaceae uncultured	HUT5UCF07IKN7B	3
Bacteria Firmicutes Clostridia Clostridiales Ruminococcaceae uncultured	HUT5UCF07H5S4Y	2
Bacteria Firmicutes Clostridia Clostridiales Ruminococcaceae uncultured	HUT5UCF07HZBAB	2
Bacteria Firmicutes Clostridia Clostridiales vadinBB60	HUT5UCF07H4A0J	3
Bacteria Firmicutes Clostridia Clostridiales vadinBB60	HUT5UCF07H4QPN	2
Bacteria Firmicutes Clostridia Clostridiales vadinBB60	HUT5UCF07IENPO	1
Bacteria Firmicutes Clostridia Clostridiales vadinBB60	HUT5UCF07HZJVZ	1
Bacteria Firmicutes Clostridia Clostridiales vadinBB60	HUT5UCF07IC8CS	1
Bacteria Firmicutes Clostridia Clostridiales vadinBB60	HUT5UCF07IC5IK	1
Bacteria Firmicutes Clostridia Clostridiales vadinBB60	HUT5UCF07IPN8Q	1
Bacteria Firmicutes Clostridia Clostridiales vadinBB60	HUT5UCF07IONZQ	1
Bacteria Firmicutes Erysipelotrichia Erysipelotrichales Erysipelotrichaceae uncultured	HUT5UCF07HYCJ2	1
Bacteria Firmicutes Negativicutes Selenomonadales Acidaminococcaceae uncultured	HUT5UCF07HZ916	1
Bacteria Gemmatimonadetes Gemmatimonadetes BD2-11_group	HUT5UCF07II59K	1
Bacteria Gemmatimonadetes Gemmatimonadetes BD2-11_group	HUT5UCF07H5NB7	1
Bacteria Lentisphaerae Lentisphaeria Victivallales Victivallaceae Victivallis	HUT5UCF07ILFAA	1
Bacteria Lentisphaerae Lentisphaeria Victivallales Victivallaceae Victivallis	HUT5UCF07H2LE2	1
Bacteria Lentisphaerae MSBL3	HUT5UCF07IBYXE	1

Bacteria Lentisphaerae Oligosphaeria Oligosphaerales	HUT5UCF07IRA34	1
Bacteria Lentisphaerae Oligosphaeria Oligosphaerales	HUT5UCF07IHUX4	1
Bacteria Lentisphaerae RFP12_gut_group	HUT5UCF07H0L32	2
Bacteria Planctomycetes vadinHA49	HUT5UCF07H606C	1
Bacteria Planctomycetes vadinHA49	HUT5UCF07IH0DW	9
Bacteria Planctomycetes vadinHA49	HUT5UCF07HYIKT	3
Bacteria Planctomycetes vadinHA49	HUT5UCF07IIE0Y	2
Bacteria Planctomycetes vadinHA49	HUT5UCF07HVSJ0	2
Bacteria Planctomycetes vadinHA49	HUT5UCF07H0FCX	2
Bacteria Planctomycetes vadinHA49	HUT5UCF07IA6IN	1
Bacteria Planctomycetes vadinHA49	HUT5UCF07ID0E0	1
Bacteria Planctomycetes vadinHA49	HUT5UCF07IQFCO	1
Bacteria Planctomycetes vadinHA49	HUT5UCF07IIB5D	1
Bacteria Planctomycetes vadinHA49	HUT5UCF07IIJP7	1
Bacteria Planctomycetes vadinHA49	HUT5UCF07IIF54	1
Bacteria Planctomycetes vadinHA49	HUT5UCF07IHWOS	1
Bacteria Planctomycetes vadinHA49	HUT5UCF07H3NT7	1
Bacteria Planctomycetes vadinHA49	HUT5UCF07H4P6A	1
Bacteria Planctomycetes vadinHA49	HUT5UCF07HXBJ1	1
Bacteria Proteobacteri aBetaproteobacteria Burkholderiales Oxalobacteraceae Herbaspirillum	HUT5UCF07H0TD4	2
Bacteria Proteobacteria Alphaproteobacteria Caulobacterales Caulobacteraceae uncultured	HUT5UCF07IOVVT	1
Bacteria Proteobacteria Alphaproteobacteria Caulobacterales Hyphomonadaceae Oceanicaulis	HUT5UCF07H8DA4	1
Bacteria Proteobacteria Alphaproteobacteria Rhizobiale sRhizobiaceae Rhizobium	HUT5UCF07IDPB0	1
Bacteria Proteobacteria Alphaproteobacteria Rhizobiales Aurantimonadaceae Martelella	HUT5UCF07IFRMO	1
Bacteria Proteobacteria Alphaproteobacteria Rhizobiales Bradyrhizobiaceae Bradyrhizobium	HUT5UCF07H5AN6	1
Bacteria Proteobacteria Alphaproteobacteria Rhizobiales Hyphomicrobiaceae Devosia	HUT5UCF07H5BHI	3
Bacteria Proteobacteria Alphaproteobacteria Rhizobiales Hyphomicrobiaceae Devosia	HUT5UCF07H4JZZ	1
Bacteria Proteobacteria Alphaproteobacteria Rhizobiales Hyphomicrobiaceae Dichotomicrobium	HUT5UCF07IQ164	1
Bacteria Proteobacteria Alphaproteobacteria Rhizobiales Hyphomicrobiaceae Hyphomicrobium	HUT5UCF07H23TK	1
Bacteria Proteobacteria Alphaproteobacteria Rhizobiales Hyphomicrobiaceae Rhodomicrobium	HUT5UCF07IBE55	1
Bacteria Proteobacteria Alphaproteobacteria Rhizobiales Hyphomicrobiaceae Rhodoplanes	HUT5UCF07H91IG	1
Bacteria Proteobacteria Alphaproteobacteria Rhizobiales Methylocystaceae Pleomorphomonas	HUT5UCF07HXZAW	1
Bacteria Proteobacteria Alphaproteobacteria Rhizobiales Rhizobiaceae Rhizobium	HUT5UCF07IJQ43	1

Bacteria Proteobacteria Alphaproteobacteria Rhizobiales Rhizobiaceae Rhizobium	HUT5UCF07H3A2C	1
Bacteria Proteobacteria Alphaproteobacteria Rhizobiales uncultured	HUT5UCF07IM3GP	1
Bacteria Proteobacteria Alphaproteobacteria Rhizobiales uncultured	HUT5UCF07IO3KM	1
Bacteria Proteobacteria Alphaproteobacteria Rhizobiales uncultured	HUT5UCF07IHVRP	1
Bacteria Proteobacteria Alphaproteobacteria Rhizobiales uncultured	HUT5UCF07H21SP	1
Bacteria Proteobacteria Alphaproteobacteria Rhizobiales Xanthobacteraceae uncultured	HUT5UCF07IOKOJ	1
Bacteria Proteobacteria Alphaproteobacteria Rhizobiales Xanthobacteraceae uncultured	HUT5UCF07H2XSZ	1
Bacteria Proteobacteria Alphaproteobacteria Rhizobiales Xanthobacteraceae uncultured	HUT5UCF07HWVBM	1
Bacteria Proteobacteria Alphaproteobacteria Rhodobacterale sRhodobacteraceae Roseovarius	HUT5UCF07ID34B	1
Bacteria Proteobacteria Alphaproteobacteria Rhodobacterales Rhodobacteraceae	HUT5UCF07H50QV	1
Bacteria Proteobacteria Alphaproteobacteria Rhodobacterales Rhodobacteraceae Citreicella	HUT5UCF07IHBX6	1
Bacteria Proteobacteria Alphaproteobacteria Rhodobacterales Rhodobacteraceae Dinoroseobacter	HUT5UCF07H4K2T	1
Bacteria Proteobacteria Alphaproteobacteria Rhodobacterales Rhodobacteraceae Donghicola	HUT5UCF07H50PI	2
Bacteria Proteobacteria Alphaproteobacteria Rhodobacterales Rhodobacteraceae Gaetbulicola	HUT5UCF07IHF3E	2
Bacteria Proteobacteria Alphaproteobacteria Rhodobacterales Rhodobacteraceae Gaetbulicola	HUT5UCF07IETPM	1
Bacteria Proteobacteria Alphaproteobacteria Rhodobacterales Rhodobacteraceae Loktanella	HUT5UCF07H6XCS	2
Bacteria Proteobacteria Alphaproteobacteria Rhodobacterales Rhodobacteraceae Loktanella	HUT5UCF07IRKWT	1
Bacteria Proteobacteria Alphaproteobacteria Rhodobacterales Rhodobacteraceae Loktanella	HUT5UCF07H984P	1
Bacteria Proteobacteria Alphaproteobacteria Rhodobacterales Rhodobacteraceae Marivita	HUT5UCF07IH7T3	1
Bacteria Proteobacteria Alphaproteobacteria Rhodobacterales Rhodobacteraceae Oceanicella	HUT5UCF07IIIVK	2
Bacteria Proteobacteria Alphaproteobacteria Rhodobacterales Rhodobacteraceae Oceanicella	HUT5UCF07IK698	1
Bacteria Proteobacteria Alphaproteobacteria Rhodobacterales Rhodobacteraceae Oceanicella	HUT5UCF07IJLMH	1
Bacteria Proteobacteria Alphaproteobacteria Rhodobacterales Rhodobacteraceae Oceanicola	HUT5UCF07HZFZ0	2
Bacteria Proteobacteria Alphaproteobacteria Rhodobacterales Rhodobacteraceae Paracoccus	HUT5UCF07H1G46	1
Bacteria Proteobacteria Alphaproteobacteria Rhodobacterales Rhodobacteraceae Ponticoccus	HUT5UCF07H51P2	1
Bacteria Proteobacteria Alphaproteobacteria Rhodobacterales Rhodobacteraceae Roseibacterium	HUT5UCF07H8STO	1
Bacteria Proteobacteria Alphaproteobacteria Rhodobacterales Rhodobacteraceae Roseicyclus	HUT5UCF07H7FLQ	1
Bacteria Proteobacteria Alphaproteobacteria Rhodobacterales Rhodobacteraceae Roseovarius	HUT5UCF07IDPYV	5
Bacteria Proteobacteria Alphaproteobacteria Rhodobacterales Rhodobacteraceae Roseovarius	HUT5UCF07HYMK9	4
Bacteria Proteobacteria Alphaproteobacteria Rhodobacterales Rhodobacteraceae Roseovarius	HUT5UCF07H73RD	4
Bacteria Proteobacteria Alphaproteobacteria Rhodobacterales Rhodobacteraceae Roseovarius	HUT5UCF07IG5UL	1
Bacteria Proteobacteria Alphaproteobacteria Rhodobacterales Rhodobacteraceae Roseovarius	HUT5UCF07IO5AC	1
Bacteria Proteobacteria Alphaproteobacteria Rhodobacterales Rhodobacteraceae Sediminimonas	HUT5UCF07IAX5N	3

Bacteria Proteobacteria Alphaproteobacteria Rhodobacterales Rhodobacteraceae Sediminimonas	HUT5UCF07IKD30	1
Bacteria Proteobacteria Alphaproteobacteria Rhodobacterales Rhodobacteraceae Sediminimonas	HUT5UCF07IK0B9	1
Bacteria Proteobacteria Alphaproteobacteria Rhodobacterales Rhodobacteraceae Thalassococcus	HUT5UCF07H88CT	1
Bacteria Proteobacteria Alphaproteobacteria Rhodobacterales Rhodobacteraceae Tropicimonas	HUT5UCF07H323H	2
Bacteria Proteobacteria Alphaproteobacteria Rhodobacterales Rhodobacteraceae Tropicimonas	HUT5UCF07H2VR5	5
Bacteria Proteobacteria Alphaproteobacteria Rhodobacterales Rhodobacteraceae Tropicimonas	HUT5UCF07ILXO8	2
Bacteria Proteobacteria Alphaproteobacteria Rhodobacterales Rhodobacteraceae Tropicimonas	HUT5UCF07IDFSX	1
Bacteria Proteobacteria Alphaproteobacteria Rhodobacterales Rhodobacteraceae Tropicimonas	HUT5UCF07H6K11	1
Bacteria Proteobacteria Alphaproteobacteria Rhodobacterales Rhodobacteraceae uncultured	HUT5UCF07IH76W	2
Bacteria Proteobacteria Alphaproteobacteria Rhodobacterales Rhodobacteraceae uncultured	HUT5UCF07IFKCD	2
Bacteria Proteobacteria Alphaproteobacteria Rhodobacterales Rhodobacteraceae uncultured	HUT5UCF07IBBVL	1
Bacteria Proteobacteria Alphaproteobacteria Rhodobacterales Rhodobacteraceae uncultured	HUT5UCF07IBMTL	1
Bacteria Proteobacteria Alphaproteobacteria Rhodobacterales Rhodobacteraceae uncultured	HUT5UCF07ID2QB	1
Bacteria Proteobacteria Alphaproteobacteria Rhodobacterales Rhodobacteraceae uncultured	HUT5UCF07INMNC	1
Bacteria Proteobacteria Alphaproteobacteria Rhodobacterales Rhodobacteraceae uncultured	HUT5UCF07INSPF	1
Bacteria Proteobacteria Alphaproteobacteria Rhodobacterales Rhodobacteraceae uncultured	HUT5UCF07IK6CJ	1
Bacteria Proteobacteria Alphaproteobacteria Rhodobacterales Rhodobacteraceae uncultured	HUT5UCF07H6LZG	1
Bacteria Proteobacteria Alphaproteobacteria Rhodospirillales Rhodospirillaceae Azospirillum	HUT5UCF07H4H5R	1
Bacteria Proteobacteria Alphaproteobacteria Rhodospirillales Rhodospirillaceae Rhodovibrio	HUT5UCF07HXA0T	1
Bacteria Proteobacteria Alphaproteobacteria Rhodospirillales Rhodospirillaceae Skermanella	HUT5UCF07H8MOZ	1
Bacteria Proteobacteria Alphaproteobacteria Rhodospirillales Rhodospirillaceae Thalassospira	HUT5UCF07H8GF9	2
Bacteria Proteobacteria Alphaproteobacteria Rhodospirillales Rhodospirillaceae Thalassospira	HUT5UCF07IBE9E	1
Bacteria Proteobacteria Alphaproteobacteria Rhodospirillales Rhodospirillaceae Thalassospira	HUT5UCF07IPA7Z	1
Bacteria Proteobacteria Alphaproteobacteria Rhodospirillales Rhodospirillaceae Thalassospira	HUT5UCF07IIR75	1
Bacteria Proteobacteria Alphaproteobacteria Rhodospirillales Rhodospirillaceae Thalassospira	HUT5UCF07IKTJD	1
Bacteria Proteobacteria Alphaproteobacteria Rhodospirillales Rhodospirillaceae uncultured	HUT5UCF07HYHDE	2
Bacteria Proteobacteria Alphaproteobacteria Rhodospirillales Rhodospirillaceae uncultured	HUT5UCF07IIXIX	1
Bacteria Proteobacteria Alphaproteobacteria Rhodospirillales Rhodospirillaceae uncultured	HUT5UCF07IC0I4	1
Bacteria Proteobacteria Alphaproteobacteria Rhodospirillales Rhodospirillaceae uncultured	HUT5UCF07H9TIA	1
Bacteria Proteobacteria Alphaproteobacteria Rhodospirillales Rhodospirillaceae uncultured	HUT5UCF07H5JTZ	1
*Bacteria Proteobacteria Alphaproteobacteria Rhodospirillales Rhodospirillales Candidatus_* Alysiosphaera	HUT5UCF07IBYWY	1
Bacteria Proteobacteria Alphaproteobacteria Rhodospirillales uncultured	HUT5UCF07H7X7Q	1
Bacteria Proteobacteria Alphaproteobacteria Rickettsiales SM2D12	HUT5UCF07IF2Y4	1

Bacteria Proteobacteria Alphaproteobacteria Rickettsiales uncultured	HUT5UCF07IA4WN	1
Bacteria Proteobacteria Alphaproteobacteria Sphingomonadales Erythrobacteraceae Erythrobacter	HUT5UCF07IAOLF	2
Bacteria Proteobacteria Alphaproteobacteria Sphingomonadales Sphingomonadaceae Novosphingobium	HUT5UCF07H5A5W	2
Bacteria Proteobacteria Alphaproteobacteria Sphingomonadales Sphingomonadaceae Novosphingobium	HUT5UCF07HZ8TV	2
Bacteria Proteobacteria Alphaproteobacteria Sphingomonadales Sphingomonadaceae Novosphingobium	HUT5UCF07H3M1S	1
Bacteria Proteobacteria Alphaproteobacteria Sphingomonadales uncultured	HUT5UCF07H3WKC	1
Bacteria Proteobacteria Betaproteobacteria Burkholderiales Burkholderiaceae Burkholderia	HUT5UCF07IBXFZ	1
Bacteria Proteobacteria Betaproteobacteria Burkholderiales Burkholderiaceae Burkholderia	HUT5UCF07IP8Y1	1
Bacteria Proteobacteria Betaproteobacteria Burkholderiales Burkholderiaceae Cupriavidus	HUT5UCF07ICC5N	1
Bacteria Proteobacteria Betaproteobacteria Burkholderiales Burkholderiaceae Pandoraea	HUT5UCF07HWNVW	5
Bacteria Proteobacteria Betaproteobacteria Burkholderiales Comamonadaceae Comamonas	HUT5UCF07IOK7Q	1
Bacteria Proteobacteria Betaproteobacteria Burkholderiales Oxalobacteraceae Undibacterium	HUT5UCF07IIDAL	1
Bacteria Proteobacteria Betaproteobacteria Rhodocyclales Rhodocyclaceae Azoarcus	HUT5UCF07IDV73	5
Bacteria Proteobacteria Betaproteobacteria Rhodocyclales Rhodocyclaceae Azoarcus	HUT5UCF07H4NRP	2
Bacteria Proteobacteria Betaproteobacteria Rhodocyclales Rhodocyclaceae Azoarcus	HUT5UCF07II479	2
Bacteria Proteobacteria Betaproteobacteria Rhodocyclales Rhodocyclaceae Azoarcus	HUT5UCF07IAILR	1
Bacteria Proteobacteria Betaproteobacteria Rhodocyclales Rhodocyclaceae Azoarcus	HUT5UCF07IC0DK	1
Bacteria Proteobacteria Betaproteobacteria Rhodocyclales Rhodocyclaceae Azoarcus	HUT5UCF07INJW5	1
Bacteria Proteobacteria Betaproteobacteria Rhodocyclales Rhodocyclaceae Azoarcus	HUT5UCF07H7DH5	1
Bacteria Proteobacteria Betaproteobacteria Rhodocyclales Rhodocyclaceae Azoarcus	HUT5UCF07HYQR6	1
Bacteria Proteobacteria Deltaproteobacteria Desulfarculales Desulfarculaceae uncultured	HUT5UCF07H2547	1
Bacteria Proteobacteria Deltaproteobacteria Desulfobacterales Desulfobacteraceae Desulfonema	HUT5UCF07HVVHB	1
Bacteria Proteobacteria Deltaproteobacteria Desulfobacterales Desulfobacteraceae Desulfosalsimonas	HUT5UCF07H36NT	1
Bacteria Proteobacteria Deltaproteobacteria Desulfobacterales Desulfobacteraceae Desulfosarcina	HUT5UCF07IBS6D	1
Bacteria Proteobacteria Deltaproteobacteria Desulfobacterales Desulfobacteraceae Desulfotignum	HUT5UCF07IGA8N	2
Bacteria Proteobacteria Deltaproteobacteria Desulfobacterales Desulfobacteraceae uncultured	HUT5UCF07H5CAA	2
Bacteria Proteobacteria Deltaproteobacteria Desulfobacterales Desulfobacteraceae uncultured	HUT5UCF07HZFGT	2
Bacteria Proteobacteria Deltaproteobacteria Desulfobacterales Desulfobacteraceae uncultured	HUT5UCF07IG9BD	1
Bacteria Proteobacteria Deltaproteobacteria Desulfobacterales Desulfobacteraceae uncultured	HUT5UCF07IAWEX	1
Bacteria Proteobacteria Deltaproteobacteria Desulfobacterales Desulfobacteraceae uncultured	HUT5UCF07ICS4G	1
Bacteria Proteobacteria Deltaproteobacteria Desulfobacterales Desulfobacteraceae uncultured	HUT5UCF07H5XZH	1
Bacteria Proteobacteria Deltaproteobacteria Desulfobacterales Desulfobulbaceae Desulfobulbus	HUT5UCF07H68GV	2
Bacteria Proteobacteria Deltaproteobacteria Desulfobacterales Desulfobulbaceae Desulfofustis	HUT5UCF07H394K	1

Bacteria Proteobacteria Deltaproteobacteria Desulfovibrionales Desulfovibrionaceae Desulfovibrio	HUT5UCF07H1O80	3
Bacteria Proteobacteria Deltaproteobacteria Desulfovibrionales Desulfovibrionaceae Desulfovibrio	HUT5UCF07H64XC	2
Bacteria Proteobacteria Deltaproteobacteria Desulfovibrionales Desulfovibrionaceae Desulfovibrio	HUT5UCF07H0HJA	2
Bacteria Proteobacteria Deltaproteobacteria Desulfovibrionales Desulfovibrionaceae Desulfovibrio	HUT5UCF07HWT9E	2
Bacteria Proteobacteria Deltaproteobacteria Desulfovibrionales Desulfovibrionaceae Desulfovibrio	HUT5UCF07IE49R	1
Bacteria Proteobacteria Deltaproteobacteria Desulfovibrionales Desulfovibrionaceae Desulfovibrio	HUT5UCF07H647Y	1
Bacteria Proteobacteria Deltaproteobacteria Desulfovibrionales Desulfovibrionaceae Desulfovibrio	HUT5UCF07H84QT	1
Bacteria Proteobacteria Deltaproteobacteria Desulfovibrionales Desulfovibrionaceae Desulfovibrio	HUT5UCF07H1CQR	1
Bacteria Proteobacteria Deltaproteobacteria Desulfovibrionales Desulfovibrionaceae Desulfovibrio	HUT5UCF07H094B	1
Bacteria Proteobacteria Deltaproteobacteria Desulfovibrionales Desulfovibrionaceae Desulfovibrio	HUT5UCF07HW7GN	1
Bacteria Proteobacteria Deltaproteobacteria Desulfovibrionales Desulfovibrionaceae Desulfovibrio	HUT5UCF07H1M9R	1
Bacteria Proteobacteria Deltaproteobacteria Desulfovibrionales Desulfovibrionaceae Desulfovibrio	HUT5UCF07H28QJ	1
Bacteria Proteobacteria Deltaproteobacteria Desulfovibrionales Desulfovibrionaceae uncultured	HUT5UCF07H0SXE	1
Bacteria Proteobacteria Deltaproteobacteria Desulfuromonadales GR-WP33-58	HUT5UCF07IEJRG	2
Bacteria Proteobacteria Deltaproteobacteria Desulfuromonadales GR-WP33-58	HUT5UCF07HVO4S	2
Bacteria Proteobacteria Deltaproteobacteria Desulfuromonadales GR-WP33-58	HUT5UCF07IG3S2	1
Bacteria Proteobacteria Deltaproteobacteria Myxococcales 0319-6G20	HUT5UCF07IEDVD	1
Bacteria Proteobacteria Deltaproteobacteria Myxococcales uncultured	HUT5UCF07H2PMD	1
Bacteria Proteobacteria Deltaproteobacteria Myxococcales uncultured	HUT5UCF07H2ABV	1
Bacteria Proteobacteria Deltaproteobacteria Rs-K70_termite_group	HUT5UCF07IB4HK	1
Bacteria Proteobacteria Deltaproteobacteria Rs-K70_termite_group	HUT5UCF07H0HOO	5
Bacteria Proteobacteria Deltaproteobacteria Rs-K70_termite_group	HUT5UCF07HZ8M6	2
Bacteria Proteobacteria Deltaproteobacteria Rs-K70_termite_group	HUT5UCF07H4APD	2
Bacteria Proteobacteria Deltaproteobacteria Rs-K70_termite_group	HUT5UCF07HX6WJ	2
Bacteria Proteobacteria Deltaproteobacteria Rs-K70_termite_group	HUT5UCF07H1P8P	2
Bacteria Proteobacteria Deltaproteobacteria Rs-K70_termite_group	HUT5UCF07IFZXK	1
Bacteria Proteobacteria Deltaproteobacteria Rs-K70_termite_group	HUT5UCF07IEGPQ	1
Bacteria Proteobacteria Deltaproteobacteria Rs-K70_termite_group	HUT5UCF07IB6R6	1
Bacteria Proteobacteria Deltaproteobacteria Rs-K70_termite_group	HUT5UCF07H4IT4	1
Bacteria Proteobacteria Deltaproteobacteria Rs-K70_termite_group	HUT5UCF07HXZA0	1
Bacteria Proteobacteria Deltaproteobacteria uncultured	HUT5UCF07ID0NB	1
Bacteria Proteobacteria Gammaproteobacteria Alteromonadales Alteromonadaceae Pseudohaliea	HUT5UCF07IQK9T	1
Bacteria Proteobacteria Gammaproteobacteria Chromatiales Chromatiaceae Thiohalocapsa	HUT5UCF07H8JKB	5

Bacteria Proteobacteria Gammaproteobacteria Chromatiales Chromatiaceae Thiohalocapsa	HUT5UCF07H73SV	2
Bacteria Proteobacteria Gammaproteobacteria Chromatiales Chromatiaceae Thiohalocapsa	HUT5UCF07INTNQ	1
Bacteria Proteobacteria Gammaproteobacteria Chromatiales Chromatiaceae Thiohalocapsa	HUT5UCF07IKDQT	1
Bacteria Proteobacteria Gammaproteobacteria Chromatiales Chromatiaceae Thiohalocapsa	HUT5UCF07HU48P	1
Bacteria Proteobacteria Gammaproteobacteria Chromatiales Chromatiaceae Thiohalocapsa	HUT5UCF07H0TW8	1
Bacteria Proteobacteria Gammaproteobacteria Chromatiales Ectothiorhodospiraceae Acidiferrobacter	HUT5UCF07IEWVM	1
Bacteria Proteobacteria Gammaproteobacteria Chromatiales Ectothiorhodospiraceae Thioalkalivibrio	HUT5UCF07IIZQY	2
Bacteria Proteobacteria Gammaproteobacteria Chromatiales Halothiobacillaceae Halothiobacillus	HUT5UCF07IOY1Y	1
Bacteria Proteobacteria Gammaproteobacteria Chromatiales Halothiobacillaceae Halothiobacillus	HUT5UCF07IQFE5	1
Bacteria Proteobacteria Gammaproteobacteria Enterobacteriales Enterobacteriaceae Escherichia-Shigella	HUT5UCF07H4NEL	1
Bacteria Proteobacteria Gammaproteobacteria Enterobacteriales Enterobacteriaceae Kluyvera	HUT5UCF07H9AKF	1
Bacteria Proteobacteria Gammaproteobacteria Legionellales Legionellaceae Legionella	HUT5UCF07H3B2T	1
Bacteria Proteobacteria Gammaproteobacteria Order_*Incertae_Sedis* Family_*Incertae_Sedis*	HUT5UCF07IBELD	2
Bacteria Proteobacteria Gammaproteobacteria Order_*Incertae_Sedis* Family_*Incertae_Sedis*	HUT5UCF07H4C4R	2
Bacteria Proteobacteria Gammaproteobacteria Order_*Incertae_Sedis* Family_*Incertae_Sedis*	HUT5UCF07IG1L0	1
Bacteria Proteobacteria Gammaproteobacteria Order_*Incertae_Sedis* Family_*Incertae_Sedis*	HUT5UCF07IG694	1
Bacteria Proteobacteria Gammaproteobacteria Order_*Incertae_Sedis* Family_*Incertae_Sedis*	HUT5UCF07INDW7	1
Bacteria Proteobacteria Gammaproteobacteria Order_*Incertae_Sedis* Family_*Incertae_Sedis*	HUT5UCF07ILI09	1
Bacteria Proteobacteria Gammaproteobacteria Order_*Incertae_Sedis* Family_*Incertae_Sedis*	HUT5UCF07ILC7C	1
Bacteria Proteobacteria Gammaproteobacteria Pseudomonadales Moraxellaceae Acinetobacter	HUT5UCF07H4JQT	1
Bacteria Proteobacteria Gammaproteobacteria Pseudomonadales Pseudomonadaceae Pseudomonas	HUT5UCF07H2S34	1
Bacteria Proteobacteria Gammaproteobacteria uncultured	HUT5UCF07HYANI	1
Bacteria Proteobacteria Gammaproteobacteria uncultured	HUT5UCF07H4IOT	1
Bacteria Proteobacteria Gammaproteobacteria uncultured	HUT5UCF07HX2WP	1
Bacteria Proteobacteria Gammaproteobacteria Xanthomonadales uncultured	HUT5UCF07INR23	1
Bacteria Proteobacteria Gammaproteobacteria Xanthomonadales uncultured	HUT5UCF07H8QXM	1
Bacteria Proteobacteria Gammaproteobacteria Xanthomonadales Xanthomonadaceae Stenotrophomonas	HUT5UCF07HZ02Z	1
Bacteria Spirochaetae Spirochaetes Spirochaetales Spirochaetaceae Spirochaeta	HUT5UCF07IJ93J	2
Bacteria Spirochaetae Spirochaetes Spirochaetales Spirochaetaceae Spirochaeta	HUT5UCF07IEB3X	1
Bacteria Spirochaetae Spirochaetes Spirochaetales Spirochaetaceae Spirochaeta	HUT5UCF07IFCCT	1
Bacteria Spirochaetae Spirochaetes Spirochaetales Spirochaetaceae Spirochaeta	HUT5UCF07IC5VL	1
Bacteria Spirochaetae Spirochaetes Spirochaetales Spirochaetaceae Spirochaeta	HUT5UCF07IMP7G	1
Bacteria Spirochaetae Spirochaetes Spirochaetales Spirochaetaceae Spirochaeta	HUT5UCF07HV7RT	1

Bacteria Spirochaetae Spirochaetes Spirochaetales Spirochaetaceae Spirochaeta	HUT5UCF07H9SM8	1
Bacteria Spirochaetae Spirochaetes Spirochaetales Spirochaetaceae Spirochaeta	HUT5UCF07H4RG3	1
Bacteria Spirochaetae Spirochaetes Spirochaetales Spirochaetaceae Spirochaeta	HUT5UCF07H868L	1
Bacteria Spirochaetae Spirochaetes Spirochaetales Spirochaetaceae Spirochaeta	HUT5UCF07H4ET1	1
Bacteria Spirochaetae Spirochaetes Spirochaetales Spirochaetaceae Treponema	HUT5UCF07HY7WN	1
Bacteria Spirochaetae Spirochaetes Spirochaetales Spirochaetaceae Treponema	HUT5UCF07IRB3L	6
Bacteria Spirochaetae Spirochaetes Spirochaetales Spirochaetaceae Treponema	HUT5UCF07H0VOE	1
Bacteria Spirochaetae Spirochaetes Spirochaetales Spirochaetaceae Treponema	HUT5UCF07HZT01	1
Bacteria Spirochaetae Spirochaetes Spirochaetales Spirochaetaceae Treponema	HUT5UCF07IRTYX	2
Bacteria Spirochaetae Spirochaetes Spirochaetales Spirochaetaceae Treponema	HUT5UCF07IPWW4	1
Bacteria Spirochaetae Spirochaetes Spirochaetales Spirochaetaceae Treponema	HUT5UCF07ILWSW	1
Bacteria Spirochaetae Spirochaetes Spirochaetales Spirochaetaceae Treponema	HUT5UCF07H320J	1
Bacteria Spirochaetae Spirochaetes Spirochaetales Spirochaetaceae Treponema	HUT5UCF07H49JP	1
*Bacteria Synergistetes Synergistia Synergistales Synergistaceae Candidatus_* Tammella	HUT5UCF07ICRAF	2
*Bacteria Synergistetes Synergistia Synergistales Synergistaceae Candidatus_* Tammella	HUT5UCF07II3KG	1
*Bacteria Synergistetes Synergistia Synergistales Synergistaceae Candidatus_* Tammella	HUT5UCF07H14PF	1
*Bacteria Synergistetes Synergistia Synergistales Synergistaceae Candidatus_* Tammella	HUT5UCF07HW6FP	1
Bacteria Synergistetes Synergistia Synergistales Synergistaceae uncultured	HUT5UCF07IM5MF	3
Bacteria Synergistetes Synergistia Synergistales Synergistaceae uncultured	HUT5UCF07H1AHV	1
Bacteria Synergistetes Synergistia Synergistales Synergistaceae uncultured	HUT5UCF07H068L	1
Bacteria Synergistetes Synergistia Synergistales Synergistaceae uncultured	HUT5UCF07H84X2	2
